# Reinforcement of STAT3 activity reprogrammes human embryonic stem cells to naive-like pluripotency

**DOI:** 10.1038/ncomms8095

**Published:** 2015-05-13

**Authors:** Hongwei Chen, Irène Aksoy, Fabrice Gonnot, Pierre Osteil, Maxime Aubry, Claire Hamela, Cloé Rognard, Arnaud Hochard, Sophie Voisin, Emeline Fontaine, Magali Mure, Marielle Afanassieff, Elouan Cleroux, Sylvain Guibert, Jiaxuan Chen, Céline Vallot, Hervé Acloque, Clémence Genthon, Cécile Donnadieu, John De Vos, Damien Sanlaville, Jean- François Guérin, Michael Weber, Lawrence W Stanton, Claire Rougeulle, Bertrand Pain, Pierre-Yves Bourillot, Pierre Savatier

**Affiliations:** 1 INSERM, U846, Stem Cell and Brain Research Institute, 18 Avenue Doyen Lépine, Bron F-69500, France; 2 Université de Lyon, Lyon 138672, France; 3 Stem Cell and Developmental Biology, Genome Institute of Singapore, Singapore 138672, Singapore; 4 INRA, USC1361, 18 Avenue Doyen Lépine, Bron F-69500, France; 5 UMR 7242 Biotechnology and Cell Signaling, University of Strasbourg, CNRS, 300 Boulevard Sébastien Brant, BP 10413, Illkirch F-67412, France; 6 UMR7216 Epigenetics and Cell Fate, CNRS, University of Paris Diderot, Sorbonne Paris Cité, Paris F-75013, France; 7 INRA, UMR1388 Génétique, Physiologie et Systèmes d'Elevage, Castanet Tolosan F-31326, France; 8 INSERM, U1040, Montpellier F-34000, France; 9 CHU Montpellier, Institute for Research in Biotherapy, Hôpital Saint-Eloi, Montpellier F-34000, France; 10 Department of Genetics, Lyon University Hospital, CNRS UMR 5292, INSERM U1028, Bron F-69500, France

## Abstract

Leukemia inhibitory factor (LIF)/STAT3 signalling is a hallmark of naive pluripotency in rodent pluripotent stem cells (PSCs), whereas fibroblast growth factor (FGF)-2 and activin/nodal signalling is required to sustain self-renewal of human PSCs in a condition referred to as the primed state. It is unknown why LIF/STAT3 signalling alone fails to sustain pluripotency in human PSCs. Here we show that the forced expression of the hormone-dependent STAT3-ER (ER, ligand-binding domain of the human oestrogen receptor) in combination with 2i/LIF and tamoxifen allows human PSCs to escape from the primed state and enter a state characterized by the activation of STAT3 target genes and long-term self-renewal in FGF2- and feeder-free conditions. These cells acquire growth properties, a gene expression profile and an epigenetic landscape closer to those described in mouse naive PSCs. Together, these results show that temporarily increasing STAT3 activity is sufficient to reprogramme human PSCs to naive-like pluripotent cells.

## Abstract (web summary)

LIF/STAT3 signalling characterizes naive pluripotency in mouse embryonic stem cells (ESCs), but whether this pathway can sustain a similar state in human cells is not completely understood. Here the authors show that LIF stimulation and enhancement of STAT3 activity allow human ESCs to escape from FGF2 dependency and facilitates their entry into a naive-like state of pluripotency.

## Graphical abstract

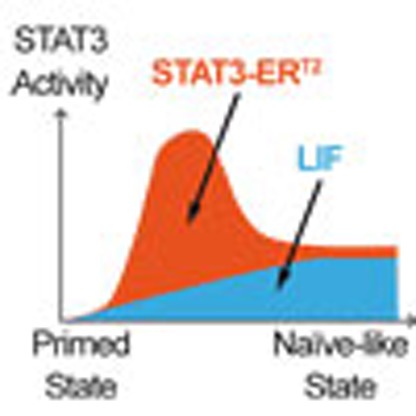

Two types of pluripotent stem cells (PSCs) have been derived from mouse embryos: (i) mouse embryonic stem cells (mESCs)^1^, which exploit leukemia inhibitory factor (LIF) signalling for self-renewal in the pluripotent state^2^, and (ii) mouse epiblast stem cells^3^,^4^, whose self-renewal capability is strictly dependent on fibroblast growth factor 2 (FGF2) and activin signalling. mESCs are derived from the early epiblast of the preimplantation embryo and are described as naive. These cells show little evidence of the expression of any lineage markers, while retaining the capacity to differentiate into any cell type. When cultured in basal medium supplemented with LIF and inhibitors of MEK and glycogen synthase kinase 3 (GSK3) signalling (2i/LIF medium), mESCs enter a new state referred to as the ground state of pluripotency^5^. The ground state reflects the state of pluripotency of the early epiblast in mouse blastocysts^6^. Although human ESCs (hESCs) are derived from preimplantation embryos, these differ in terms of growth factor requirements and gene expression profiles^7^. They are dependent on FGF2 and transforming growth factor-β (TGFβ)/activin/nodal signalling for differentiation inhibition^8^ and they do not express markers of naive/ground-state pluripotency as defined in rodents. Similar to EpiSCs derived from the late epiblast of the mouse post-implantation embryo^3^,^4^, they express early lineage markers, which is a characteristics features of the so-called primed pluripotency.

The generation of hESC lines with growth requirements and self-renewal properties comparable to those of mESCs remains a challenge. Several groups have described culture conditions for generating hESCs that share various properties with mESCs^9^,^10^,^11^,^12^,^13^,^14^,^15^. Hanna *et al.*^9^ pioneered the field by demonstrating that two transcription factor cocktails, Klf2 and Klf4 on one hand and Oct4 and Klf4 on the other, were capable of reprogramming hESCs and human induced PSCs (hiPSCs) towards naive-like pluripotency. In this work, the transition from primed to naive pluripotency was exemplified by the *de novo* capacity of the reprogrammed cells to self-renew in the 2i/LIF medium. However, following transgene shutdown, the reprogrammed cells ceased self-renewal after 20 passages, suggesting that transgenes had retained these cells in the naive state but were not stably reprogrammed. Gafni *et al.*^10^ resolved this problem by culturing transgene-free hESCs and hiPSCs in 2i/LIF medium supplemented with FGF2 and TGFβ1, in combination with pharmacological inhibitors of JNK and p38^MAPK^ (the so-called NHSM condition). The resulting cell lines could be regularly passaged in a naive-like state with no apparent limits. Chan *et al.*^11^ developed a similar approach using mTesr1 medium supplemented with LIF and MEK, GSK3β and BMP4 inhibitors (the so-called 3iL condition), to drive hESCs into a pluripotency state that resembles a preimplantation epiblast. In two other studies, naive-like pluripotency was obtained using knockout serum replacement (KOSR) supplemented with FGF2, MEK and GSK3β inhibitors^12^, or FGF2, MEK, GSK3β and ROCK inhibitors^13^. Notably, all four reports used FGF2 in propagating hESCs, whereas two of studies employed FGF2 and TGFβ1 (refs 10, 11), which are growth factors associated with primed pluripotency^8^. Theunissen *et al.*^14^ used N2B27 basal medium supplemented with inhibitors of MEK, GSK3β, ROCK, SRC and BRAF kinases, together with LIF and activin, to establish FGF2-independent naive hESC lines (the so-called 5i/A/L condition). The 5i/A/L condition captured a distinct pluripotent state in humans, which closely resembles that of mESCs at the transcriptome level. However, the 5i/A/L cells were not dependent on LIF/JAK for self-renewal. In the most recent study, Takashima *et al.*^15^showed that a short-term expression of two components, NANOG and KLF2, was sufficient to ignite other elements of the network and reset the human pluripotent state. Inhibition of ERK and protein kinase C sustained a transgene-independent rewired state.

Murine ESCs exploit the LIF/JAK signalling pathway and the transcription factor signal transducer and activator of transcription 3 (STAT3) to self-renew in the pluripotent state^16^,^17^,^18^. STAT3 can sustain self-renewal without the addition of LIF when it is overexpressed in the form of a hormone-dependent STAT3 that is directly activated by estradiol^19^,^20^. Endogenous STAT3 and ligand-dependent STAT3 activate the transcription of a common set of genes that include those that encode transcription factors (*Klf4*, *Klf5*, *Smad7*, *Tfcp2L1*, *c-fos*, *JunB*, *Zfp36*, *Zfp36L1*, *Gbx2*, *Sp5*, *Sall4* and *c-myc*), serine/threonine kinases (*Pim1*, *Pim3* and *Sgk*), inhibitors of signalling pathways (*Socs2* and *Socs3*) and adhesion molecules (*Icam1* and *Ocln*). These genes are involved in blocking the process of differentiation^19^,^21^,^22^,^23^. Furthermore, *Klf4* and *Tfcp2L1* have been shown to revert mouse epiblast stem cells into mESCs when overexpressed^23^,^24^. The key role of STAT3 in naive pluripotency is reinforced by the observation that LIF-JAK/STAT3 signalling is a limiting factor for reprogramming to naive pluripotency^25^, and that JAK/STAT3 signalling can be sufficient and dominant over FGF/ERK signalling, thus enabling the induction of a naive pluripotent state^26^. All these data prompted us to examine the capacity of STAT3 to confer LIF dependency to hESCs and to reprogramme these towards naive pluripotency. We demonstrated that transient exogenous STAT3 activity, in combination with LIF stimulation, allows hESCs to escape from FGF2 dependency and, on treatment with MEK and GSK3β inhibitors, facilitates their entry into a new state, designated as TL2i, with genetic and epigenetic characteristics of naive pluripotency.

## Results

### Enhancement of STAT3 activity and regulation of its targets

We started by establishing a new male hESC line from a human supernumerary embryo, designated as OSCAR. Characterization of the OSCAR cell line is presented in Supplementary Fig. 1. OSCAR cells were infected with the simian immunodeficiency virus (SIV)-based lentiviral vector GAE-STAT3-ER^T2^ expressing a hormone-dependent mouse STAT3 driven by a CAG promoter^19^. One clone, designated as F-OS3–10 (F designates FGF2 dependency), was selected for further analysis. Two other clones were produced after infecting the female hESC line H9 with the GAE-STAT3-ER^T2^ lentiviral vector, designated as F-H9S3-2 and F-H9S3–14.

**Figure 1:**
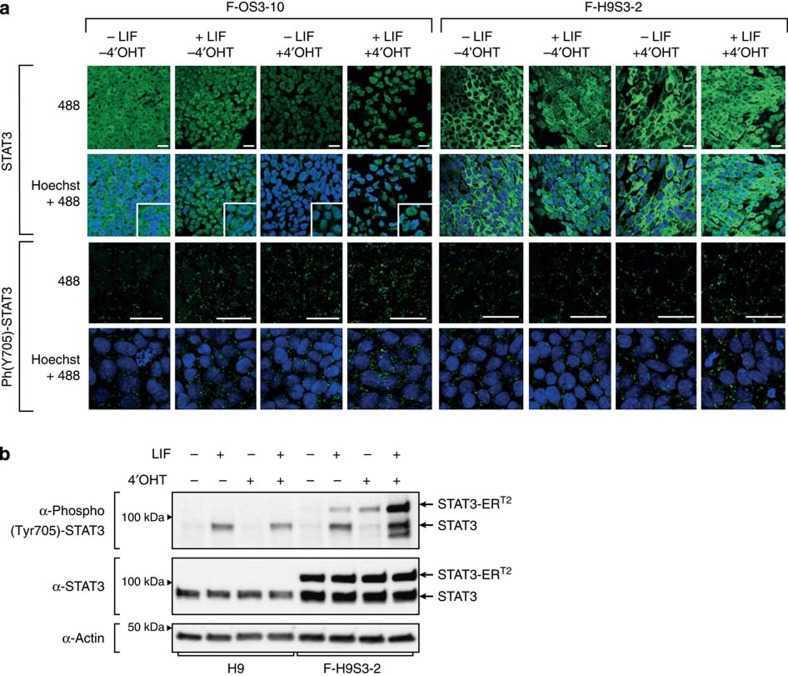
Reinforcement of STAT3 activity in F-OS3 and F-H9S3 cells. (**a**) Immunofluorescence labelling of F-OS3–10 and F-H9S3-2 cells with antibodies to STAT3 and phospho-STAT3 (Tyr705), without stimulation of LIF and 4′-OHT (−LIF −4′-OHT), after stimulation with LIF (+LIF −4′-OHT), with 4′-OHT (−LIF +4′-OHT) and with both (+LIF+4′-OHT). Scale bar, 30 μm. One representative experiment of two repeats is shown. (**b**) Western blot analysis of STAT3, phospho-(Tyr705)-STAT3 expression in H9 and F-H9S3-2 cells after stimulation with 10,000 U ml^−1^ LIF, 250 nM 4′-OHT, or both for 1 h. One representative experiment of three repeats is shown.

We analysed the subcellular localization of STAT3 by immunolabelling. Stimulation of F-OS3–10 for 1 h, either with 10,000 U ml^−1^ human LIF or with 250 nM 4-hydroxytamoxifen (4′-OHT), induced nuclear translocation of STAT3, as previously described in mESCs^20^. Complete translocation was only observed when cells were treated with both LIF and 4′-OHT (Fig. 1a). The yield of STAT3 phosphorylation on tyrosine-705 showed a similar response to LIF and 4′-OHT treatment; after stimulation with each one of these two molecules, only rare cells exhibited nuclear staining, whereas the nuclear expression of phospho-STAT3 was considerably increased in cells treated with both. Stimulation of F-H9S3-2 cells showed similar results. Stimulation with 4′-OHT induced STAT3 translocation in some cells, whereas LIF stimulation induced partial translocation of STAT3 in most cells. As described in the F-OS3–10 cells, nuclear staining for STAT3 was strongly reinforced when F-H9S3-2 cells were stimulated with both LIF and 4′-OHT. Contrary to the observations involving F-OS3–10 cells, nuclear localization of phospho-STAT3 was induced by 4′-OHT and was only marginally increased after further stimulation with LIF.

To discriminate between endogenous STAT3 and STAT3-ER^T2^, the level of STAT3 phosphorylation was examined by western blotting in F-H9S3 and control cells (Fig. 1b and Supplementary Fig. 2). 4′-OHT induced phosphorylation of STAT3-ER^T2^, whereas LIF induced phosphorylation of both STAT3 and STAT3-ER^T2^ on tyrosine-705, as previously described in mESCs. The expression of phospho-(Tyr705)-STAT3 was considerably increased in cells treated with both LIF and 4′-OHT, further demonstrating the synergistic effect of the two molecules. This conclusion was reinforced by the analysis of STAT3 target gene expression, which showed that some target genes were only activated on stimulation with both LIF and 4′-OHT, suggesting that the two factors act synergistically to regulate the transcription of STAT3 target genes (Supplementary Fig. 3a). Intriguingly, we observed a slight increase in the level of STAT3-ER^T2^ in the +LIF/4′-OHT condition. We speculate that it might result from the gradual selection of cells with the highest STAT3-ER^T2^ activity during propagation in culture. The expression of phosphorylated-(Ser727)-STAT3 was examined in Supplementary Figs 3b and 2.

### Self-renewal induced by LIF and hormone-dependent STAT3

**Figure 2:**
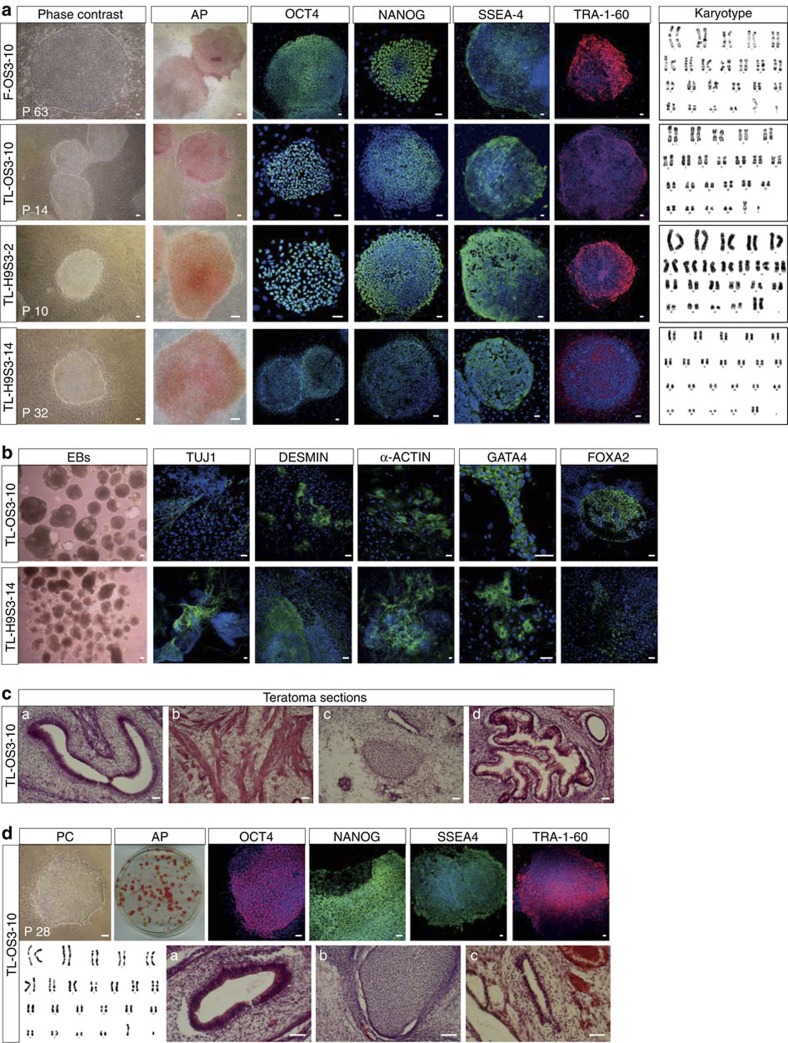
LIF and hormone-dependent STAT3 sustain self-renewal and maintain pluripotency of TL-OS3 and TL-H9S3 cells. (**a**) Characterization of F-OS3–10, TL-OS3–10, TL-H9S3-2 and TL-H9S3–14 cell lines by phase-contrast microphotography, AP staining, G-band karyotyping and immunofluorescence labelling with antibodies to OCT4, NANOG, TRA-1–60 and SSEA4. G-band karyotyping was performed on P39 (F-OS3–10), P12 (TL-OS3–10) and P5 (TL-H9S3-2 and TL-H9S3–14). (**b**) Immunofluorescence labelling of TL-OS3–10 and TL-H9S3–14 cells with antibodies to TUJ1 (neurons), DESMIN (muscle), α-ACTIN (heart), FOXA2 and GATA4 (endoderm) after differentiation as induced by the formation of EBs. (**c**) Teratoma formation with the TL-OS3–10 cells. Haematoxylin and eosin (H&E) staining showing neurectodermal (**a**), cartilage (**b**), muscle (**c**) and gut-like structures (**d**) (*n*=6 testes). (**d**) Characteristics of TL-OS3–10 cells after propagation on gelatin without MEF; PC, phase-contrast microphotograph of an undifferentiated colony; AP, alkaline phosphatase activity; immunofluorescence labelling with antibodies to OCT4, NANOG, SSEA4 and TRA-1–60; karyotype at P41; teratoma sections showing neuroepithelium (**a**), cartilage (**b**) and gland structure (**c**). Scale bar, 50 μm (*n*=4 testes).

Based on the observation that treatment of STAT3-ER^T2^-expressing cells with both LIF and 4′-OHT induced massive phosphorylation of STAT3-ER^T2^ and activated the expression of several key targets of STAT3 activity, we asked whether LIF and 4′-OHT could sustain self-renewal. To address this, FGF2 was withdrawn from the culture medium of F-OS3–10, F-H9S3-2 and F-H9S3–14 cells, and replaced with LIF and 250 nM 4′-OHT. Cells treated with only 4′-OHT or LIF differentiated (Supplementary Fig. 4), in accordance with the results of a previous report that showed that neither LIF nor a constitutively active STAT3 was sufficient in maintaining hESCs in an undifferentiated state^27^. In contrast, when the three lines were treated with the two factors for two to three passages, they gradually adapted to the new growth conditions and ultimately formed large colonies typical of undifferentiated cells (Fig. 2a). These colonies expressed alkaline phosphatase (AP), transcription factors OCT4 and NANOG, stage-specific embryonic antigen (SSEA)-4 and TRA-1–60 cell surface markers. These new cells were called TL-OS3–10, TL-H9S3-2 and TL-H9S3–14 cells, respectively (TL designates 4′-OHT+LIF dependency). For each cell line, a normal chromosome complement was observed after 20 passages. TL-OS3–10 and TL-H9S3–14 cells were induced to differentiate in a suspension culture (embryoïd bodies). Both cell lines retained the capacity to differentiate into derivatives of the ectoderm (TUJ1^+^), mesoderm (DESMIN^+^ and α-ACTIN^+^) and endoderm (FOXA2^+^ and GATA4^+^) (Fig. 2b). Moreover, they were capable of forming teratomas that contained derivatives of the three germ layers after injection into the testes of severe combined immunodeficient (SCID) mice (Fig. 2c and Supplementary Fig. 5). These new cells thus expressed the cardinal markers and properties of pluripotency. TL cells have been cultured up to 70 passages by mechanical dissociation. When the TL-OS3–10 cells were transferred back to a culture medium lacking LIF and 4′-OHT, and supplemented with 5 ng ml^−1^ FGF2, these reverted to an FGF2-dependent state designated as Reverted (R)-OS3–10 cells (Supplementary Fig. 6).

The TL-OS3–10 cells readily adapted to growth on gelatin in 20% KOSR. They formed undifferentiated colonies that were positive for AP activity, as well as OCT4, NANOG, SSEA4 and TRA-1–60 expression (Fig. 2d). They demonstrated a normal 46,XY karyotype after 41 passages on gelatin and could form teratomas with derivatives of the three germ layers. These findings indicate that LIF and hormone-dependent STAT3 sustain self-renewal and maintain the pluripotency of hESCs.

### LIF and STAT3-ER^T2^ overcome FGF2-dependency

**Figure 3:**
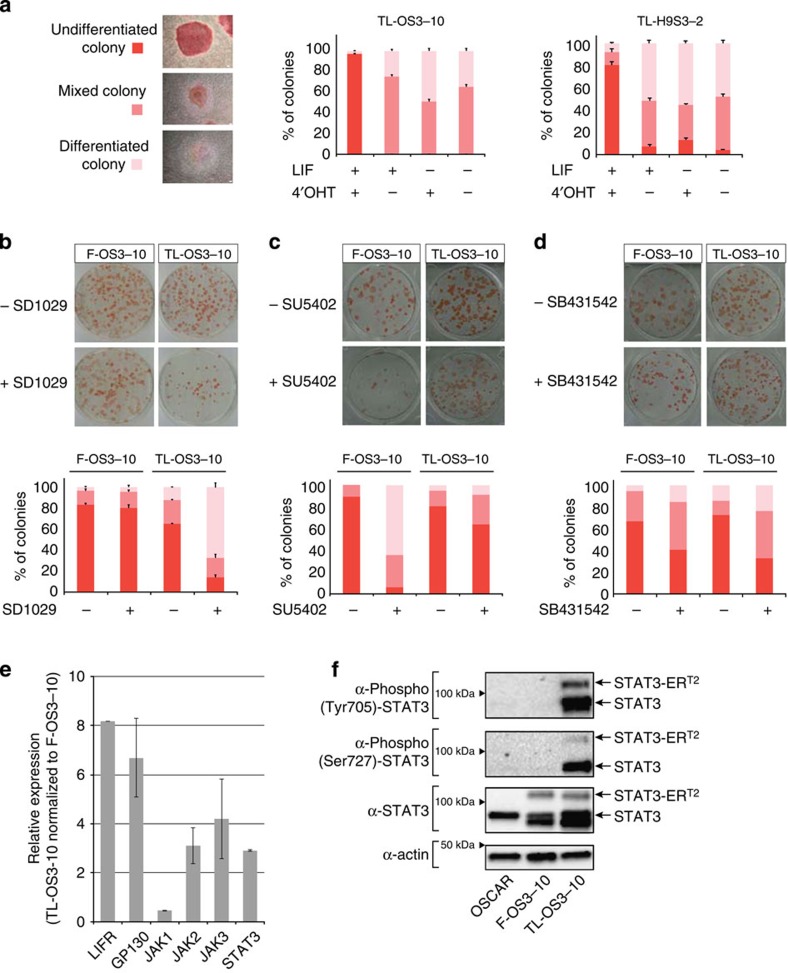
Signalling pathways in TL cells. (**a**) A colony-forming assay with TL-OS3–10 and TL-H9S3-2 cells. Cell clumps were plated in a medium supplemented with LIF, 4′-OHT, or both, and cultivated for 5 days. After staining to reveal AP activity, the colonies were scored and the percentage of undifferentiated, mixed and differentiated colonies was calculated. *n*=3; error bars indicate the mean ±s.d. (**b**–**d**) The colony-forming assay with F-OS3–10 and TL-OS3–10 cells (representative experiment). Cell clumps were plated in a medium supplemented with pharmacological inhibitors of JAK2 (SD1029 at 10 μM), FGFR (SU5402 at 25 μM) and SMADs (SB431542 at 10 μM), and cultivated for 5 days with FGF2 (F-OS3–10) and 3 days with LIF+4′-OHT (TL-OS3–10). Upper panels: staining to reveal AP activity; Bottom panels: histograms showing the percentage of undifferentiated, mixed and differentiated colonies (*n*=3; error bars indicate the mean±s.e.m.). (**e**) Reverse transcriptase–qPCR analysis of LIFR, GP130, JAK and STAT3 expression in TL-OS3–10 versus F-OS3–10 cells. (**f**) Western blot analysis of STAT3 and STAT3-ER^T2^ expression in OSCAR, F-OS3–10 and TL-OS3–10 cells, analysed with antibodies to total STAT3, phospho-(Tyr705)-STAT3 and phospho-(Ser727)-STAT3. One representative experiment of three repeats is shown.

All attempts to adapt F-OS3–10 cells in a medium supplemented with either factors (LIF or 4′-OHT) were unsuccessful (Supplementary Fig. 4), suggesting that LIF and 4′-OHT act in synergy to sustain self-renewal of TL-OS3–10 cells. To explore this further, the dependency of TL cells for LIF and 4′-OHT was assessed using a colony-forming assay (Fig. 3a). TL-OS3–10 and TL-H9S3-2 cells were dissociated into small clumps, plated at a low density on feeder cells and further grown for 5 days in a medium supplemented with 10,000 U ml^−1^ LIF, 250 nM 4′-OHT, or both. The resulting colonies were stained for AP activity and the percentage of undifferentiated, mixed and differentiated colonies was calculated. In the presence of the two factors, the percentage of undifferentiated colonies in the TL-OS3–10 cells increased to 97%. On withdrawal of LIF or of 4′-OHT, all colonies differentiated. In addition, the withdrawal of both factors resulted in similar percentages of mixed and differentiated colonies. We concluded that TL cells were dependent on both LIF and 4′-OHT for differentiation blockade.

To gain some further insight into the signalling pathways that sustain self-renewal, pharmacological inhibitors of JAK2 (SD1029), FGF receptor (SU5402) and activin receptor (SB431542) were applied to F-OS3–10 and TL-OS3–10 cells for 5 days. Their capacity to self-renew or to differentiate was assessed using the colony-forming assay (Fig. 3b–d). On treatment with SD1029 for 5 days, the F-OS3–10 cells mostly formed undifferentiated colonies, whereas the TL-OS3–10 cells subjected to the same conditions formed mixed and differentiated colonies (Fig. 3b). This observation indicated that TL-OS3–10 cells were dependent on JAK kinase for self-renewal and survival. In contrast, the TL-OS3–10 cells showed higher percentages of undifferentiated colonies after treatment with SU5402, as compared with the F-OS3–10 cells, which indicated that TL cells were relieved of their dependency on FGF receptor (FGFR) signalling (Fig. 3c). A reduction in the percentage of undifferentiated colonies was consistently observed on treatment of both F-OS3–10 and TL-OS3–10 cells with SB431542 compared with untreated cells, suggesting that dependency on activin signalling was not completely abolished in TL cells (Fig. 3d).

To gain some insight into the activity of the LIF signalling pathway in the TL cells, the expression of its elements was assessed. The levels of LIF receptor (LIFR), GP130, JAKs and STAT3 all increased in TL-OS3–10 cells compared with that in F-OS3–10 cells (Fig. 3e). The level of phospho-(Tyr705)-STAT3 and phospho-(Ser727)-STAT3 was considerably higher in TL cells compared with the control cells (Fig. 3f and Supplementary Fig. 2). We concluded that reprogramming of F to TL state and the capacity of TL cells to sustain LIF-dependent self-renewal are associated with global activation of the LIF signalling pathway.

### Activation of STAT3 target genes in TL cells

**Figure 4:**
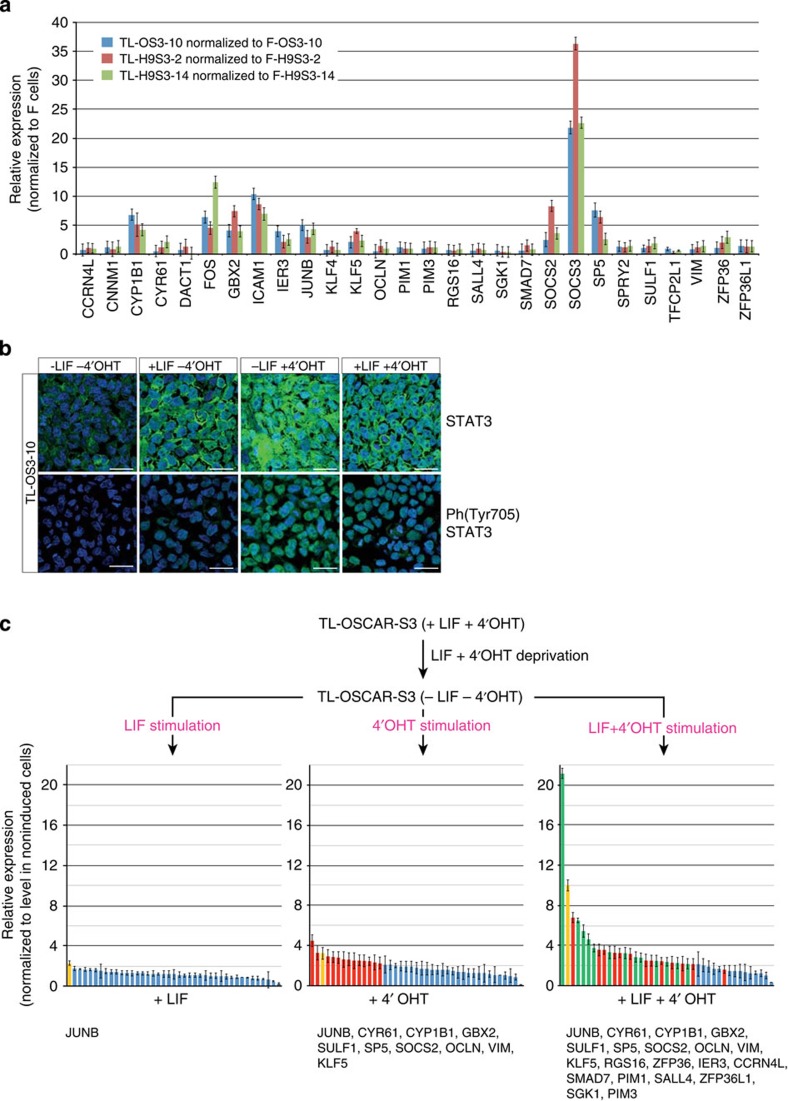
Activation of STAT3 target genes in TL cells. (**a**) Histogram representation of the messenger RNA level (ΔCt) of STAT3 target genes in F-OS3–10, F-H9S3-2, F-H9S3–14, TL-OS3–10, TL-H9S3-2 and TL-H9S3–14 after normalization to β*-actin* (ΔCt=1; *n*=3, mean±s.d.). (**b**) Immunofluorescence labelling of TL-OS3–10 cells with antibodies to STAT3 and phospho-(Y705)-STAT3, after LIF and 4′-OHT starvation (−LIF −4′-OHT) and after stimulation with LIF (+LIF −4′-OHT), with 4′-OHT (−LIF +4′-OHT) and with both (+LIF +4′-OHT). Scale bar, 30 μm. One representative experiment of two repeats is shown. (**c**) Histogram representation of the mRNA level (ΔCt) of STAT3 target genes in TL-OS3–10 cells after LIF+4′-OHT starvation for 24 h and re-stimulation with 10,000 U ml^−1^ LIF, 250 nM 4′-OHT, or both for 2 h. One gene, shown in yellow, is activated by LIF stimulation (>2-fold). Following stimulation with 4′-OHT, 13 additional genes (shown in red) are activated (>2-fold). Following stimulation with both LIF and 4′-OHT, 13 additional genes (shown in green) are activated (>2-fold). All ΔCt were normalized to *GAPDH* (ΔCt=1; *n*=3, mean±s.d.).

To gain insight into the mechanism by which TL cells acquired dependency on LIF and 4′-OHT for self-renewal, we analysed the phosphorylation and nuclear translocation of STAT3, and the expression of STAT3 target genes. Only ten STAT3 target genes were continuously upregulated in TL-OS3 cells (*CYP1B1*, *C-FOS*, *ICAM1*, *IER3*, *JUNB*, *GBX2*, *KLF5*, *SOCS2*, *SOCS3* and *SP5*) (Fig. 4a). All of these STAT3 targets were repressed on reversion of TL-OS3 to FGF2-dependent R-OS3 cells. These genes have been shown to participate in the blockade of differentiation in mESCs^19^,^21^,^28^,^29^,^30^,^31^. The same STAT3 target genes were activated in TL-H9S3-2 and TL-H9S3–14 clones compared with the F-H9S3-2 and F-H9S3–14 cells, respectively. Notably, the other STAT3 targets identified in mESCs were not continuously activated in TL cells.

It was then determined whether the acute activation of STAT3 target genes required both LIF and 4′-OHT. To address this, TL-OS3–10 cells were deprived of LIF and 4′-OHT for 16 h and re-stimulated with LIF, 4′-OHT, or both. Immunolabelling showed abundant nuclear translocation of STAT3 and phospho-(Y705)-STAT3 after stimulation with 4′-OHT or with 4′-OHT and LIF (Fig. 4b). Quantitative reverse transcriptase–PCR analysis of STAT3 target gene expression showed that only one gene showed at least a twofold induction on stimulation with LIF, 14 genes showed a 2- to 4.5- fold induction with 4′-OHT and 27 genes showed a 2- to 21-fold activation with both LIF and 4′-OHT (Fig. 4c). Thus, LIF and 4′-OHT act synergistically to activate the transcription of STAT3 target genes in TL cells. It is quite striking, however, that *KLF4* was not activated in any of these two experiments, despite its key role as a STAT3 target gene in mESCs^19^,^32^,^33^,^34^. This is in agreement with a previous observation that forced Stat3 activation in mouse EpiSCs is insufficient for Klf4 induction^35^.

### Signalling pathway reconfiguration

**Figure 5:**
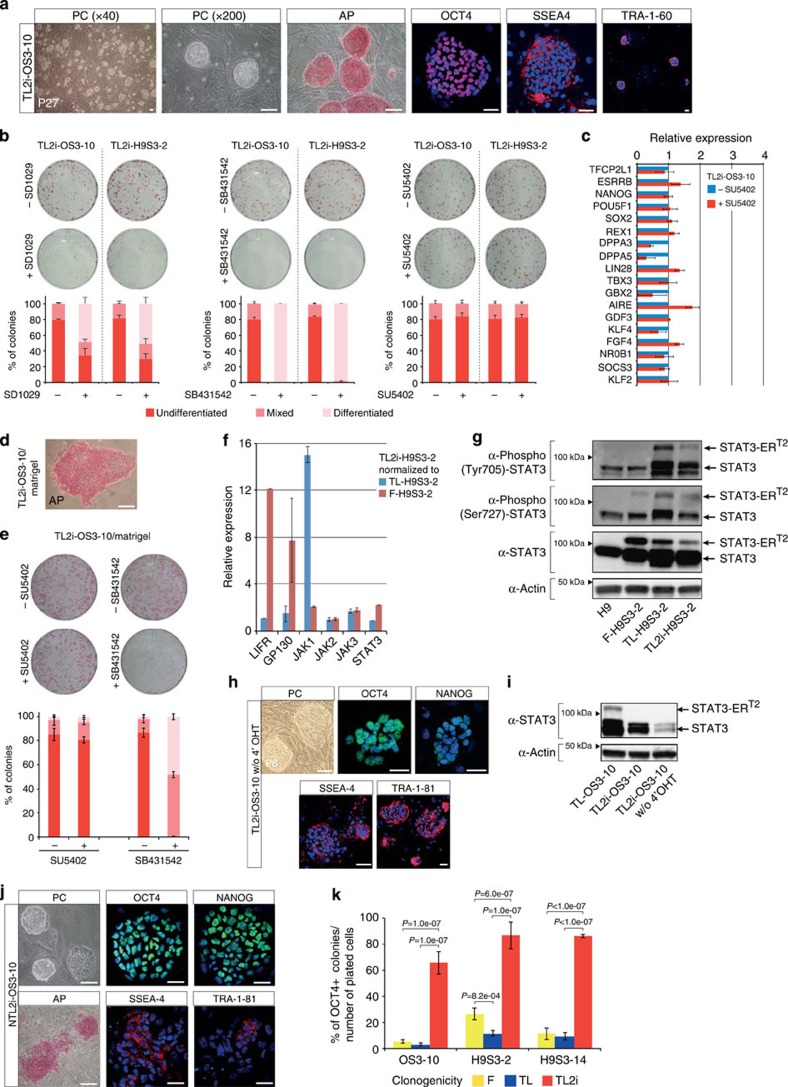
Signalling pathways in TL2i cells. (**a**) Phase-contrast microphotographs, AP activity and immunofluorescence labelling of OCT4, SSEA4 and TRA-1–60 in TL2i cells. (**b**) A colony-forming assay with TL2i-OS3–10 and TL2i-H9S3-2 cells (representative experiment). Cell clumps were plated in TL2i medium supplemented with pharmacological inhibitors of JAK2 (SD1029 at 10 μM), FGFR (SU5402 at 10 μM) and SMADs (SB431542 at 10 μM), and cultivated for 5 days. Upper panels: staining to reveal AP activity; bottom panels: histograms showing the percentage of undifferentiated, mixed and differentiated colonies. (*n*=3; error bars indicates the mean±s.e.m.). (**c**) Histogram representation of the mRNA level (ΔCt) of pluripotency genes in TL2i-OS3–10 cells before and treatment with FGFR inhibitor SU5402 for 5 days after normalization to *GAPDH* (ΔCt=1). (*n*=3, mean±s.d.). (**d**) Characteristics of TL2i-OS3–10 cells after propagation on Matrigel without MEF; AP, alkaline phosphatase activity. (**e**) Histograms showing the percentage of undifferentiated, mixed and differentiated colonies (*n*=3; error bars indicate the mean±s.e.m.) in a colony-forming assay with TL2i-OS3–10 cells (representative experiment). Cell clumps were plated in a medium supplemented with pharmacological inhibitors of FGFR (SU5402 at 10 μM) and SMADs (SB431542 at 10 μM), and cultivated for 5 days with LIF+4′-OHT. (**f**) Histogram representation of the mRNA level (ΔCt) of *LIFR*, *GP130*, *JAK* and *STAT3* genes in TL2i-H9S3-2 cells, after normalization to *GAPDH* (ΔCt=1), then to TL-H9S3-2 (blue bars) and F-H9S3-2 cells (red bars). (*n*=3, mean±s. d.). (**g**) Western blot analysis of STAT3 and STAT3-ER^T2^ expression in OSCAR, F-H9S3-2, TL-H9S3-2 and TL2i-H9S3-2 cells, analysed with antibodies to total STAT3, phospho-(Tyr705)-STAT3 and phospho-(Ser720)-STAT3. One representative experiment of three repeats is shown. (**h**) Phase-contrast microphotographs (PC) and immunofluorescence labelling of OCT4, NANOG, SSEA4 and TRA-1–81 in TL2i-OS3–10 cells after culturing in 2i/LIF medium without 4′-OHT for 30 passages. (**i**) Western blot analysis of STAT3 and STAT3-ER^T2^ expression in TL-OS3–10 cells and TL2i-OS3–10 cells (+/−4′-OHT), analysed with antibodies to total STAT3. One representative experiment of two repeats is shown. (**j**) Phase-contrast microphotographs (PC), AP detection and immunofluorescence labelling of OCT4, NANOG, TRA-1–81 and SSEA4 in TL2i-OS3–10 cells after culturing in N2B27+2i/LIF basal medium for eight passages. (**k**) Histogram representation of the cloning efficiency of F, TL and TL2i cells (OS3–10, H9S3–2 and H9S3–14 lines) after single-cell dissociation with 0.05% trypsin-EDTA and re-plating on feeders in the presence of 10 μM ROCK inhibitor Y-27632 for 24 h post dissociation. Tukey's test; *n*=3; error bars indicate the mean±s. e. Scale bar, 50 μm (**a**,**d**,**h**,**i**).

A key feature of mESCs is the dispensability for ERK signalling to self-renewal^5^,^36^. Therefore, we asked whether ERK signalling became dispensable to TL cells. To address this, TL-OS3–10 cells were cultivated in medium supplemented with 20% KOSR, 1 μM PD325901 (MEK inhibitor), 3 μM CHIR99021 (GSK3 inhibitor), LIF and 4′-OHT (2i/LIF medium). TL cells formed tight dome-shaped colonies, which could be readily expanded for 50 passages without noticeable changes in their morphology (Fig. 5a). These TL colonies were positive for AP, OCT4, SSEA4 and TRA-1–60. These new cells were designated as TL2i-OS3–10. When cultured on non-adherent dishes, TL2i-OS3–10 cells formed embryoïd bodies, which contained cells positive for the expression of TUJ1, α-ACTIN, FOXA2 and GATA4, indicating their differentiation into ectoderm, mesoderm and endoderm (Supplementary Fig. 7b). They retained a normal karyotype over 25 passages and formed teratomas comprising three germ-layer lineages (Supplementary Fig. 7c,d). Similar results were obtained with TL-H9S3-2 and TL-H9S3–14 cells, which could be converted into TL2i-H9S3-2, and TL2i-H9S3–14 cells, respectively (Supplementary Fig. 7a,c).

To gain further insight into the signalling pathways that sustain self-renewal of TL2i cells, pharmacological inhibitors of JAK2 (SD1029), FGF receptor (SU5402) and activin receptor (SB431542) were applied to TL2i-OS3–10 and TL2i-H9S3-2 cells for 5 days. Their capacity to self-renew or to differentiate was assessed using the colony-forming assay (Fig. 5b). On treatment with SD1029 and SB431542 for 5 days, the TL2i cells mostly formed mixed and differentiated (AP^−^) colonies. This observation indicated that TL2i cells are dependent on JAK kinase and the activin receptor for self-renewal and survival. In contrast, the TL2i cells resulted in similar percentages of undifferentiated colonies after treatment with SU5402 compared with the control cells. Moreover, minimal variations in the RNA levels of pluripotency markers were observed after SU5402 treatment (Fig. 5c). Similar results were obtained with the TL2i-OS3–10 cells propagated in feeder-free conditions. These could be cultivated on Matrigel in 20% KOSR without noticeable differentiation, forming undifferentiated colonies that tested positive for AP activity (Fig. 5d). Treatment with the activin receptor inhibitor SB431542 for 5 days reduced the number of AP^+^ colonies in a colony-forming assay, suggesting that TL2i-OS3 cells still rely on Smad signalling for the maintenance of the pluripotency (Fig. 5e). By contrast, TL2i-OS3–10 cells could be cultivated in the presence of FGFR inhibitor SU5402, with no alteration in the cloning efficiency of AP^+^ cells. To further demonstrate that the TL2i cells no longer required FGF2 signalling for self-renewal in the undifferentiated state, the TL2i-H9S3-2 cells were propagated for six passages (24 days) in the presence of SU5402. They expressed OCT4, NANOG and SSEA4, and retained the capacity to form teratomas containing derivatives of the three germ layers after grafting into the testes in SCID mice (Supplementary Fig. 7e,f). Altogether, these results indicated that the TL2i cells were relieved of their dependency on FGFR signalling.

Next, we examined the expression of LIFR, gp130, JAK kinases and phospho-(Tyr705)-STAT3 in the F-H9S3-2, TL-H9S3-2 and TL2i-H9S3-2 cells. All members (LIFR, GP130, JAKs and STAT3) of the LIF/STAT3 signalling pathway were upregulated in the TL2i-H9S3-2 cells when compared with both F-H9S3-2 and TL-H9S3-2 cells (Fig. 5f). Phosphorylation of STAT3 and STAT3-ER^T2^ on Tyr705 in TL-H9S3-2 and TL2i-H9S3-2 cells was dramatically increased as compared with F cells (Fig. 5g and Supplementary Fig. 2), which coincided with the results observed in TL-OS3 cells (see Fig. 3f). However, a reduction in both total STAT3-ER^T2^ and the phospho-(Y705)-STAT3 level was also observed during the TL-H9S3-2 to TL2i-H9S3-2 transition. A possible explanation for this is that the inhibition of differentiation-promoting MEK activity of TL2i cells by PD0325901 would reduce the amount of phospho-(Y705)-STAT3 required to sustain pluripotency. This latter observation prompted us to examine the capacity of the TL2i cells to sustain propagation in the absence of exogenous STAT3 activity. Thus, the TL2i cells were cultivated in medium lacking 4′-OHT, to inactivate STAT3-ER^T2^. The TL2i cells continued to proliferate in the undifferentiated state for more than 30 passages, as evidenced by the expression of OCT4, NANOG, SSEA4 and TRA-1–81 (Fig. 5h). Surprisingly, the *STAT3-ER*^*T2*^ transgene was completely silenced in TL2i-OS3 cells (Fig. 5i and Supplementary Fig. 2). These results indicated that once established, the TL2i cells could be propagated in the absence of detectable exogenous STAT3 activity. They also suggest that STAT3-ER^T2^-expressing cells were gradually eliminated from the population after the expression of the transgene became unnecessary for self-renewal. Moreover, after injection into the testes of SCID mice, the 4′-OHT-deprived TL2i cells were capable of forming teratomas with ectodermal, mesodermal and endodermal derivatives, thus demonstrating their pluripotency (Supplementary Fig. 7g). TL2i cells could also be propagated in N2B27 basal medium supplemented with LIF for eight passages. They retained the expression of pluripotency markers (Fig. 5j).

The TL2i cells could be routinely passaged by trypsin dissociation into a single-cell suspension for more than 25 (TL2i-H9S3) and 50 passages (TL2i-OS3). Moreover, the plating efficiency of TL2i cells was augmented compared with the FGF2-dependent hESCs (from 1.5- to 20-fold) and TL cells (from 4- to 15-fold) (Fig. 5k). Up to 87% of the TL2i cells formed undifferentiated colonies after single-cell dissociation and replating. Thus, the TL2i cells have acquired permissiveness to single-cell dissociation.

### Transcriptome reconfiguration towards naive pluripotency

**Figure 6:**
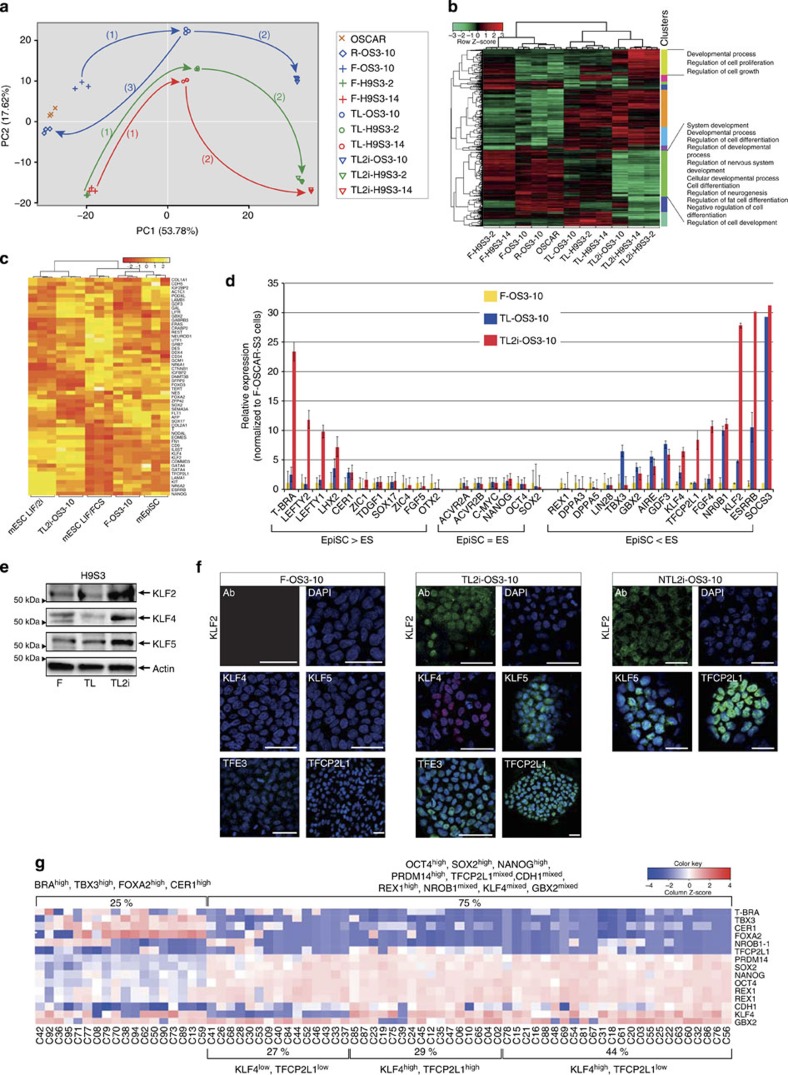
Transcriptome reconfiguration towards naive pluripotency. (**a**) Graphical representation of the first principal component of PCA for OSCAR, F-OS3–10, F-H9S3-2, F-H9S3–14, TL-OS3–10, TL-H9S3-2, TL-H9S3–14, TL2i-OS3–10, TL2i-H9S3-2, and TL2i-H9S3–14 populations based on the transcriptome data; (1) conversion from F to TL state; (2) conversion from TL to TL2i state; (3) conversion from TL to R state. (**b**) Hierarchical clustering and heatmap of transcriptome data (mean values/cell category most differentially expressed 1,000 probe sets) using Pearson correlation coefficient as a measure of distance between rows and Spearman correlation coefficient as a measure of distance between columns. (**c**) Non-supervised cross-species comparison of the transcriptome of mESCs cultivated in conventional medium (FCS+LIF) and 2i/LIF medium, mouse EpiSCs, F-OS3–10 and TL2i-OS3–10 cells. (**d**) Histogram representation of the mRNA level (ΔCt) of pluripotency and lineage marker genes in F-OS3–10, TL-OS3–10 and TL2i-OS3–10 after normalization to *GAPDH* (ΔCt=1). EpiSC>ES indicates genes that are overexpressed in mouse epiblast stem cells (mEpiSCs) compared with mESCs; EpiSC=ES indicates genes that are equally expressed in mEpiSCs and mESCs; EpiSC<ES indicates genes that are overexpressed in mESCs compared with mEpiSCs. (*n*=3, mean±s.d.). (**e**) Western blot analysis of KLF2, KLF4 and KLF5 expression in TL2i-H9S3-2 cells. One representative experiment of two repeats is shown. (**f**) Immunofluorescence labelling of F-OS3–10 and TL2i-OS3–10 cells with antibodies to KLF2, KLF4, KLF5, TFE3 and TFCP2L1, and NTL2i-OS3–10 cells with antibodies to KLF2, KLF5 and TFCP2L1. (**g**) Representative heatmap of normalized Fluidigm data for TL2i-OS3–10 cells. Each column corresponds to a single cell, whereas rows correspond to genes. Blue and red gradations highlight downregulated and upregulated genes, respectively, in relation to a random pool of TL2i-OS3–10 cells.

To characterize the transcriptomic changes that occurred during the adaptation of FGF2-dependent hESCs to TL and TL2i cells, the gene expression profile of all the cell lines generated in our study were characterized using Human Genome U219 (Affymetrix) arrays. A principal component analysis (PCA) calculated from all probe sets showed that all FGF2-dependent cell lines (parental OSCAR cells, F-OS3-10, F-H9S3-2 and F-H9S3–14) clustered together on the first axis, which represents 53% of total variability (Fig. 6a). The R-OS3-10 cells were grouped to the same cluster, which indicated that after its reversal to FGF2-dependency, the TL-OS3–10 cells recovered for the most part the gene expression profile of the original F-OS3–10 cells. All TL cells clustered together, with the two TL-H9S3 clones (2 and 14) showing the highest similarity. All TL2i cells also clustered together. Therefore, growth factor dependency was the main source of variability among the 33 samples analysed. Similarly, within-cell type and different between-growth factor dependency was supported by hierarchical clustering of the most differentially expressed 1,000 probe sets (Fig. 6b and Supplementary Table 1). Gene Ontology (GO) analysis highlighted ‘regulation of cell growth' and ‘regulation of cell proliferation' as two key cellular functions activated in TL2i cells versus all other cell types.

We next determined whether the expression of naive pluripotency markers was upregulated in the TL2i cells. To address this, we first conducted a non-supervised cross-species comparison of the transcriptomes of EpiSCs (GSE7866 (ref. 3)) and mESCs cultivated in conventional medium (FCS+LIF) and in 2i/LIF medium (GSE43421 (ref. 5)), with the human compendium. Analysis of an independent panel of genes selected by the International Stem Cell Initiative^37^ showed that the TL2i cells and ground-state mESCs formed a distinct cluster characterized by the robust expression of naive pluripotency markers, including *KLF2*, *KLF4*, *TFCP2L1* and *GBX2*, compared with mESCs cultivated in conventional medium, FGF2-dependent hESCs and EpiSCs (Fig. 6c). These results were confirmed and extended in a quantitative PCR (qPCR) analysis of naive and primed pluripotency markers in F, TL and TL2i cells. Several genes whose expression was associated with naive pluripotency in mice were dramatically upregulated in the TL2i-OS3 cells, such as *NROB1*, *GDF3*, *KLF2*, *KLF4*, *AIRE*, *GBX2*, *TFCP2L1*, *ESRRB*, *FGF4* and *TBX3* (Fig. 6d and Supplementary Fig. 8a). Four of these genes (*AIRE*, *GBX2*, *GDF3* and *NROB1*) were already upregulated in TL-OS3 cells, whereas the other six (*KLF2*, *KLF4*, *TFCP2L1*, *ESRRB*, *FGF4* and *TBX3*) were further upregulated in the TL2i-OS3 cells. Some genes whose expression was associated with primed pluripotency (*BRA*, *LEFTY1*, *LEFTY2*, *LHX2*, *CER1* and *FOXA2*) were also activated in the TL2i-OS3 cells, although the absolute expression level remained relatively low as compared with the expression level of the naive markers (Supplementary Fig. 8a). Similar results were obtained with the TL2i-H9S3 cells (Supplementary Fig. 8b,c). Notably, the expression of some of these genes was also activated after the treatment of OSCAR and H9 cells with 4′-OHT for four passages (21 days), albeit at much reduced levels (less than fourfold) compared with the activation observed in TL2i cells versus F cells (Supplementary Fig. 8d). Thus, we concluded that activation of naive markers in TL2i-OS3 cells resulted from cell reprogramming by STAT3-ER^T2^ and LIF, and not from the direct effect of 4′-OHT on the transcriptional regulation of naive pluripotency genes. All TL2i cell lines were clustered close to ground-state mESCs (Supplementary Fig. 9 and Supplementary Table 2). The activation of naive state-specific transcription factors in TL2i cells was confirmed by western blotting (KLF2, KLF4 and KLF5) (Fig. 6e and Supplementary Fig. 2) and immunolabelling (KLF2, KLF4, KLF5, TFE3 and TFCP2L1) (Fig. 6f).

To evaluate the possible heterogeneity of the TL2i cell population, the expression of the previously mentioned genes was analysed in the TL2i-OS3–10 cells by single-cell qPCR. PCA analysis showed that only 9 genes out of the 35 analysed, namely *CDH1*, *TFCP2L1*, *LHX2*, *NROB1*, *BRA*, *AIRE*, *CER1*, *TBX3* and *FOXA2*, were responsible for most of the variations across individual cells (Supplementary Fig. 10a). The 73 cells analysed were classified into two main categories. Eighteen cells (25%) expressed the lowest levels of the pluripotency markers *OCT4* (*POU5F1*), *SOX2* and *NANOG*, and of the naive markers *PRDM14*, *GDF3*, *TFCP2L1*, *CDH1 (E-CADHERIN)* and *REX1* compared with the mean population (Fig. 6g and Supplementary Fig. 10b,c). The expression of primed pluripotency markers *BRA*, *TBX3*, *FOXA2* and *CER1* was higher compared with the rest of the population, although heterogeneity was also observed. These cells probably represented cells that were spontaneously committing to differentiation. On the other hand, 55 cells (75%) expressed *PRDM14*, *OCT4* (*POU5F1*), *SOX2*, *NANOG*, *CDH1* and *REX1* at higher levels and *BRA*, *TBX3*, *FOXA2* and *CER1* at lower levels compared with the core pool of PSCs. *KLF4* and *GBX2* were also heterogeneously expressed in these cells, although a large majority of the cells displayed increased expression compared with the mean population. The cells within this population might possibly represent the core pool of PSCs in the TL2i cell population. This latter population could be divided into three subpopulations based on the expression level of the naive markers *KLF4* and *TFCP2L1.* These three subpopulations exhibited the following phenotypes, *KLF4*^*low*^ and *TFCP2L1*^*low*^ (27%), *KLF4*^*high*^ and *TFCP2L1*^*low*^ (44%), and *KLF4*^*high*^ and *TFCP2L1*^*high*^ (29%).

hiPSCs were successfully reprogrammed to TL2i state, using the same protocol (Supplementary Fig. 11).

### Epigenetic reorganization

**Figure 7:**
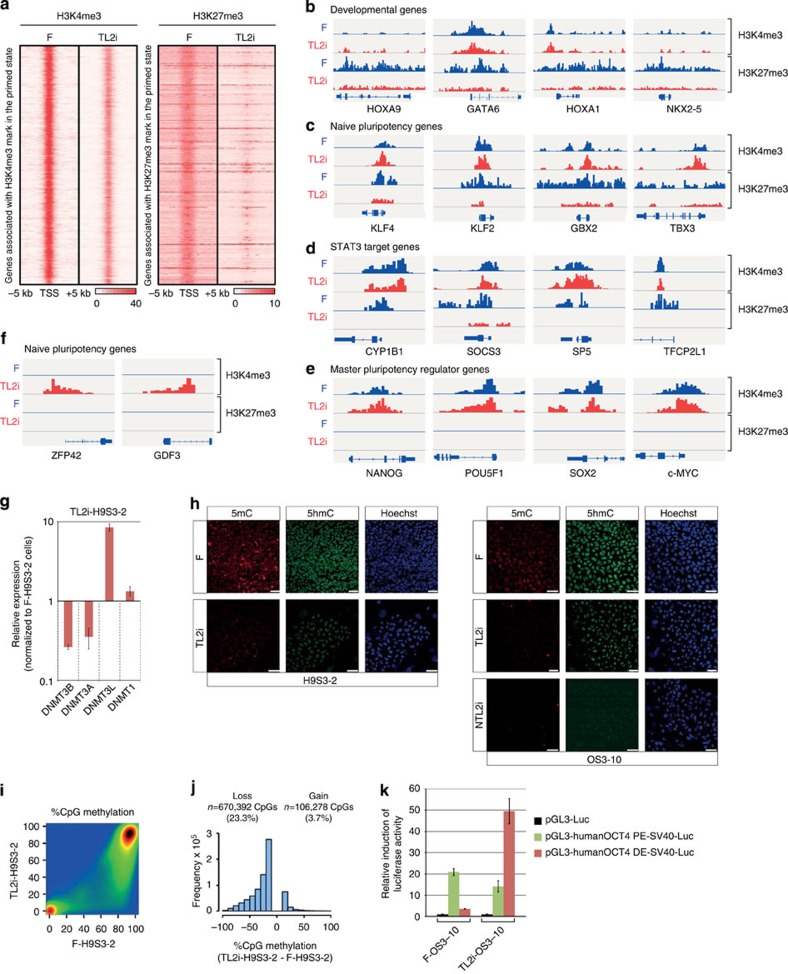
Epigenetic reorganization. (**a**) Density maps of H3K4me3 (left) and H3K27me3 (right) at loci identified as bivalent in primed hESCs (F-H9S3) and TL2i-H9S3 hESCs. (**b**–**f**) Chromatin landscape of (**b**) bivalent developmental genes *HOXA9*, *GATA6*, *HOXA1* and *NKX2-5*. (**c**) Naive pluripotency genes *KLF4*, *KLF2*, *GBX2* and *TBX3*. (**d**) STAT3 target genes *CYP1B1*, *SOCS3*, *SP5* and *TFCP2L1*. (**e**) Core pluripotency factors *NANOG*, *POU5F1*, *SOX2* and *MYC* that harbour a decrease in H3K27me3 repressive marks in TL2i-H9S3 versus F-H9S3 cells. (**f**) Naive pluripotency genes *REX1* (*ZFP42*) and *GDF3* that harbour an increase of H3K4me3 mark in TL2i-H9S3 versus F-H9S3 cells. (**g**) Histogram representation of the mRNA level (ΔCt) of DNA methyltransferases *DNMT3A*, *DNMT3B*, *DNMT3L* and *DNMT1* in TL2i-H9S3 versus F-H9S3 after normalization to *GAPDH* (ΔCt=1). (*n*=3, mean±s.d.). (**h**) Immunofluorescence labelling of F-H9S3, TL-H9S3, TL2i-H9S3, F-OS3, TL-OS3, TL2i-OS3 and TL2i-OS3 cells after propagation in N2B27 medium supplemented with 2i/LIF and 4′-OHT, with antibodies to 5mC and 5hmC. (**i**) Comparison of CpG methylation measured by RRBS in TL2i-H9S3-2 versus F-H9S3-2 cells. The graph shows a pairwise comparison of CpG methylation calculated in 400 bp tiles containing at least three CpGs with more than 8 × sequencing depth in both samples. The density of points increases from blue to dark red. (**j**) Density histogram showing the number of CpGs that lose or gain >10% methylation in TL2i-H9S3-2 compared with F-H9S3-2 cells. The figures above the graph indicate the number of CpGs and percentages compared with the total number of sampled CpGs (*n*=2,879,485). Sampled CpGs (23.3%) lose >10% methylation in TL2i-H9S3-2 versus F-H9S3-2 cells, whereas only 3.7% gain methylation. (**k**) Activity of the distal and proximal enhancer elements of the *OCT4* promoter in TL2i-OS3–10 versus F-OS3–10 cells measured in a transient expression assay with the *pGL3-humanOCT4 DE-SV40-Luc* and *pGL3-human OCT4 PE-SV40-Luc* reporter plasmids. The histogram represents luciferase activity measured in the indicated cell types after normalization to *Renilla* luciferase activity and to firefly luciferase activity measured with the *pGL3* empty plasmid (control). (*n*=3, mean±s.d.).

Conversion from primed to naive pluripotency is usually associated with an important rearrangement of active and repressive histone marks across the genome^10^,^14^. To address whether epigenomic rearrangement occurred during the reprogramming of TL2i cells, we determined the distribution of active H3K4me3 and repressive H3K27me3 chromatin marks in both F-H9S3-2 and TL2i-H9S3-2 cell lines by chromatin immunoprecipitation (ChIP) sequencing. Consistent with previously published data on human naive ESCs^10^,^14^, both H3K4me3 and, more importantly, H3K27me3 signals were strongly reduced in TL2i cells compared with that observed in F cells at the transcriptional start site of genes harbouring bivalent marks (Fig. 7a). More specifically, we observed a global reduction of H3K27me3 signal in the TL2i-H9S3-2 cells at (i) developmental genes that carry bivalent domains, including *HOXA9*, *GATA6*, *HOXA1* and *NKX2.5* (Fig. 7b); (ii) genes that are specific of the naive state of pluripotency in mESCs, including *KLF2*, *KLF4*, *GBX2* and *TBX3* (Fig. 7c); and (iii) STAT3 target genes *CYP1B1*, *SOCS3*, *SP5* and *TFCP2L1* (Fig. 7d). Interestingly, we also observed a slight increase in the H3K4 me3 signal for *KLF4* and *GBX2* (Fig. 7d,e), which corroborates with their upregulation in TL2i cells. The core pluripotency transcription factors, including *NANOG*, *OCT4*, *SOX2* and *MYC* exclusively harboured the H3K4me3 active mark in both TL2i and F cells (Fig. 7e). Interestingly, for some markers of ground-state pluripotency such as *REX1* and *GDF3*, we observed a gain of the H3K4me3 signal during conversion from F to TL2i pluripotency state (Fig. 7f). Together, these data indicate that the conversion from F to TL2i state is associated with an important rearrangement of active and repressive histone marks at the genome-wide level. Moreover, the gain of H3K4me3 and loss of H3K27me3 at specific regulators of naive state pluripotency and STAT3 target genes correlate with transcriptional data observed in F and TL2i cells.

We analysed the expression levels of DNA methyl-transferases using quantitative reverse transcriptase–PCR in TL2i cells and observed no alteration in *DNMT1* expression, decrease in *DNMT3A* and *DNMT3B* expression, and increase in *DNMT3L* expression compared with that observed in F cells (Fig. 7g), which is concordant with the results of previous studies^10^,^14^. We also analysed the global levels of 5-methylcytosine (5mC) and 5-hydroxy-mC (5hmC), and found that the transition from the F to TL2i state is accompanied by a decrease in global 5mC and 5hmC levels, consistent with previous observations in mouse naive ESCs^38^,^39^,^40^ (Fig. 7h). TL2i cells propagated in N2B27 medium for eight passages exhibited a further reduction in 5mC and 5hmC levels. To better quantify genome methylation, we generated single-base profiles of cytosine methylation by reduced representation bisulfite sequencing (RRBS) for approximately three million CpGs in F-H9S3-2 and TL2i-H9S3-2 cells, and observed 23% decrease in global DNA methylation (Fig. 7i,j). The reduction of methylation in TL2i-H9S3-2 cells is reminiscent, but to a lesser extent, of the global erasure of methylation in mouse naive stem cells cultured in 2i conditions^39^,^40^,^41^.

We examined the activity of the proximal and distal enhancers (PE and DE, respectively) of the OCT4 promoter in TL2i cells. PE is equally active in primed and naive pluripotency states, whereas DE is minimally active in the primed state and strongly active in the naive state^3^,^14^,^42^,^43^. Expression plasmids carrying the luciferase under the transcriptional control of the distal element DE and proximal element PE^10^ were transfected into F and TL2i cells. A 14-fold increase in luciferase activity was observed in the TL2i-OS3 cells transfected with the DE-luciferase compared with that of F-OS3 cells, revealing a much stronger activity of the DE element in the reprogrammed cells (Fig. 7k).

Naive pluripotency in mice is characterized by the presence of two active X chromosomes in female cells, both *in vivo* and *in vitro*. To assess the activity of the X chromosomes, we analysed the expression of *XIST*, which coats the completely inactive X chromosome (Xi); *ATRX*, which is expressed only by the active X chromosome (Xa); and *XACT*, which is a long non-coding RNA that coats both Xa and partially reactivated Xi (that is, Xi*)^44^. The TL2i-H9S3-2 cells did not show *XIST* expression in the RNA-fluorescent *in situ* hybridization (FISH) study, indicating the absence of Xi chromosome (Supplementary Fig. 12). However, we observed the presence of a single *ATRX* pinpoint and bi-allelic *XACT* clouds, indicating the presence of Xi*, as previously reported^41^,^45^,^46^. Thus, we concluded that the second X chromosome is partially inactive in the TL2i-H9S3 cells.

### Transcriptome reconfiguration towards human embryo

**Figure 8:**
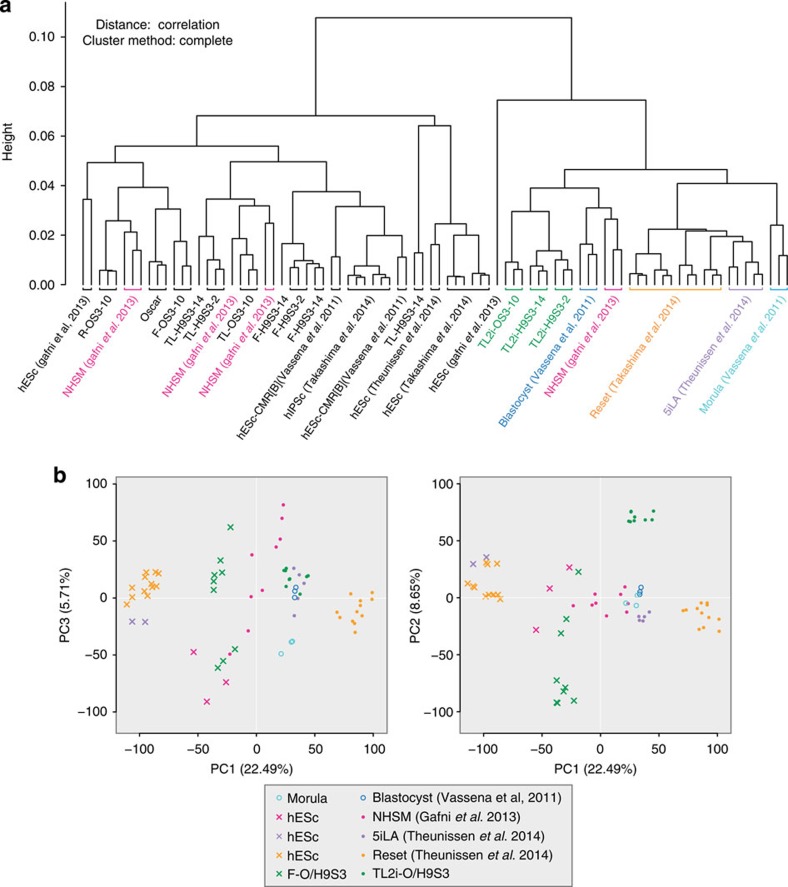
Transcriptome reconfiguration towards human embryo. (**a**) Correlation clustering of transcriptome data for OSCAR, F-OS3–10, F-H9S3-2, F-H9S3–14, TL-OS3–10, TL-H9S3-2, TL-H9S3–14, TL2i-OS3–10, TL2i-H9S3–2, TL2i-H9S3–14, NHSM hESCs and hiPSCs^10^, 5i/L/A hESCs^14^, Reset hESCs^15^, hESc-CMR[B]^47^, human morula and blastocyst^47^. (**b**) Graphical representation of PCA calculated from the aforementioned compendium. Left panel: axis 1 and 3; right panel axis 1 and 2.

We performed correlation clustering of all expressed probe sets in F, TL and TL2i cell lines (this study), and in primed and naive hESCs and hiPSCs described in previous reports^10^,^14^,^15^. To measure the distance between each cell line and human preimplantation embryo, transcriptome data from human morulae and blastocysts obtained by Vassena *et al.*^47^ (GSE29397) were included in the comparison. The TL2i cell lines, Reset cell lines^15^, 5i/L/A cell lines^14^, three of nine NHSM cell lines^10^, morulae and blastocysts were clustered together (Fig. 8a). All the primed hESCs and six other NHSM cell lines formed a distinct cluster. The proximity of the TL2i cells with the embryo samples was confirmed using PCA analysis (Fig. 8b). The TL2i cells, 5i/L/A cells and embryo samples were clustered closely together on the first and third axes of the PCA (representing 22.5% and 5.7% of the total variation, respectively). The second axis (8.6% of the total variation) differentiated the TL2i cells from the 5i/L/A cells and embryo samples. The NHSM cell lines were strongly dispersed on both the first and third axes, which was in line with the results of the correlation clustering described above. Interestingly, contrary to the F-OS3 and F-H9S3 cells, the TL2i-OS3 and TL2i-H9S3 cells were clustered closely, suggesting that reprogramming of primed hESCs to TL2i cells erased the existing differences among various cell lines and homogenized their transcriptome. Hierarchical clustering of the most differentially expressed 1,000 probe sets highlighted a cluster of upregulated genes in all naive hESCs, morulae and blastocysts (Supplementary Fig. 13 and Supplementary Table 3). GO analysis showed that this cluster was strongly enriched in genes involved in protein localization to the mitochondria, which was in line with previous results showing mitochondrial activation after the reprogramming of hESCs to naive pluripotency^15^.

## Discussion

In this study, we show that enforced STAT3 activity combined with LIF and 2i allows hESCs to escape from FGF2 dependency and to self-renew in a LIF-dependent state designated as TL2i. This is the first demonstration that the reinforcement of STAT3 activity is sufficient to elicit reprogramming of hESCs to LIF dependency. Chan *et al.*^11^ showed that culture in 3i (MEK, GSK3β and BMP4 inhibitors) elicited responsiveness of hESCs to LIF and the acquisition of LIF dependency was associated with an increased expression of the signal transducer GP130. In the present study, the reinforcement of STAT3 activity is the trigger for initial phenotypic changes, including the activation of GP130, LIFR and JAK expression, before further reprogramming by the addition of 2i.

A striking observation is the synergy between LIF and exogenous STAT3 activity for inducing LIF responsiveness in hESCs. Such a synergy between LIF and exogenous STAT3 activity for reprogramming mouse EpiSCs to ground-state pluripotency has been reported^25^. In this previous study, provision of LIF and 4′-OHT to EpiSCs expressing STAT3-ER^T2^ resulted in an extensive phosphorylation of STAT3-ER^T2^ on Tyrosine 705, a strong increase in *Socs3* expression and the formation of Oct4^+^ colonies when cells were subjected to 2i/LIF/4′-OHT conditions. Moreover, contrary to the original EpiSCs, the Oct4^+^ colonies were capable of forming chimeras after injection into a blastocyst, indicating that they had acquired the cardinal features of ground/naive-state pluripotency^25^. Here we demonstrated that a similar synergy is necessary to induce extensive phosphorylation of STAT3-ER^T2^ on Tyrosine 705, to activate the expression of most STAT3 target genes and promote self-renewal of hESCs in the absence of FGF2 in a LIF-dependent state designated as TL. LIF and 4′-OHT alone (that is, in the absence of MEK and GSK3β inhibitors) sustained long-term propagation of TL cells that harboured a mixed naive/primed phenotype: they activated the expression of naive pluripotency biomarkers; however, they still required mechanical passaging and exhibited low clonogenicity similar to FGF2-dependent hESCs. Further treatment with MEK and GSK3β inhibitors is necessary for TL cells to progress towards a naive-like state, designated as TL2i. Intriguingly, the yield of STAT3-ER^T2^ phosphorylation was strongly reduced in TL2i-H9 cells compared with TL-H9 cells, and it was completely abolished in the TL2i-OS3 cells. Moreover, TL2i cells were permissive to long-term propagation in the absence of 4′-OHT, resulting in the inactivation of the *STAT3-ER^T2^* transgene. Together, these finding suggest that once reprogramming is achieved, the level of STAT3 activity, required to maintain pluripotency, could be lowered. Consequently, exogenous STAT3-ER^T2^ activity was no longer required and the subsequent loss of selection pressure resulted in a gradual disappearance of STAT3-ER^T2^ expression. This observation is in line with that of Yang *et al.*^25^, which showed that a relatively short burst of JAK/STAT3 activation is sufficient for 2i to elicit EpiSC reprogramming.

The TL2i cells exhibited most criteria associated with the naive pluripotency state, including dome-shaped morphology of the colonies, permissiveness to single-cell dissociation, growth in the N2B27 medium, expression of mESC-specific transcription factors, loss of dependency on FGF2 signalling and gain of dependency on LIF/JAK signalling. The TL2i cells are unique with respect to the latter two criteria. None of the recently reported naive hESCs are independent of FGF2 signalling and dependent on JAK signalling for self-renewal^10^,^11^,^12^,^13^,^14^,^15^. In mice, switch from FGF2 to LIF/JAK dependency is the hallmark of the transition between primed and naive pluripotency^24^,^48^. Interestingly, *ESRRB* transcripts were detected in the TL2i cells, unlike the Reset cells^15^. The TL2i cells showed a global reduction in repressive histone marks at transcriptional start sites and a global demethylation of the genome. The yield of DNA demethylation (23%) was in line with that reported in a previous report by Gafni *et al.*^10^, which showed similar reduction in NHSM cells that was relatively modest compared with Reset cells (50%)^15^. Whether the observed difference between the TL2i, NHSM and Reset cells is meaningful with respect to the assessment of the naive status of these three types of cells is unclear. Indeed, it must be highlighted that the level of DNA methylation is higher in the human inner cell mass (ICM) (∼40%)^49^ than in the mouse ICM (∼30%)^39^, suggesting that a relatively modest reduction in the DNA methylation level across the genome is sufficient to diagnose reprogramming to *bona fide* naive pluripotency in hESCs. The TL2i cells did not exhibit reactivation of the second X chromosome. This observation was in line with the situation observed in the 5i/L/A cells, in which only one chromosome was active^14^, but was in contrast to the situation observed in the Reset cells, in which both the X chromosomes were active^15^. Both the X chromosomes are active in the human ICM^50^; however, the timing of X-chromosome inactivation (XCI) is unknown. Therefore, the discrepancy in XCI observed among the TL2i cells, 5i/L/A cells, Reset cells and NHSM-naive hESCs might be explained by the fact that these cells represent different phases of pluripotency.

The TL2i cells described in the present study showed a gene expression profile similar to that of the 5i/L/A cells^14^ and Reset cells^15^. However, the three cell types formed distinct clusters, with gene expression profiles of all these cells being closely correlated to that of the human preimplantation embryo. The reason for the observed difference in the global gene expression profiles of the three cell types is unclear. It could result from the differences in the reprogramming strategy or from variations in the epigenetic landscape between original hESC lines, or both. A key question is whether these three cell types reflect different metastable states that coexist in the human ICM and early epiblast.

## Methods

### Cell culture

All hESCs were routinely cultured at 37 °C in 5% CO_2_, 5% O_2_ in a medium containing knockout DMEM, 20% KOSR, 1 mM glutamine, 0.1 mM β-mercaptoethanol (Sigma) and 1% non-essential amino acid (NEAA, Gibco) on growth-inactivated murine embryonic fibroblasts (MEF) or 0.2% gelatin-coated dishes (for TL-OS3–10 cells only). For OSCAR, H9, F-OS3, F-H9S3 and R-OS3 cells, the complete ESC medium was supplemented with 4–8 ng ml^−1^ of FGF2. The culture medium of the TL-OS3 and TL-H9S3 cells was supplemented with 10,000 U ml^−1^ recombinant human LIF and 250 nM 4′-OHT (Calbiochem), unless otherwise indicated. For TL2i-OS3 and TL2i-H9S3 cells, the culture medium was supplemented with 10,000 U ml^−1^ hLIF, 250 nM 4′-OHT, 3 μM CHIR99021 and 1 μM PD325901 (Stemgent). OSCAR, H9, F- and R-hESCs, and TL cells were manually split and passaged every 6 days. TL2i cells were regularly passaged by single-cell dissociation with 0.05% trypsin-EDTA (Gibco) every 4 days. N2B27 basal medium was generated by inclusion of the following: DMEM/F12 (Invitrogen, 11320; 48% v/v), Neurobasal (Invitrogen, 21103; 48% v/v), N2 supplement (Invitrogen, 17502048; 1% v/v), B27 supplement (Invitrogen, 17504044; 2% v/v), 1 mM glutamine (Invitrogen), 1% NEAAs (Invitrogen), 0.1 mM β-mercaptoethanol (Sigma), penicillin–streptomycin (Invitrogen) and 50 μg ml^−1^ BSA (Sigma).

### Preparation of plasmid construct and DNA transfection

*pPCAGIZ* plasmid^19^ was digested with EcoRI, and the resulting 3.3-kbp fragment containing the entire *STAT3-ER*^*T2*^ coding sequence was subcloned between the BamHI and EcoRV sites in *pGAE-CAG-eGFP/WPRE*^51^, to generate *pGAE-STAT3-ER*^*T2*^.

For luciferase assay, 7.5 μg of *pGL3* reporter plasmids (*pGL3-humanOCT4 DE-SV40-Luc*, Addgene 52414; *pGL3-human OCT4 PE-SV40-Luc*, Addgene 52415; *pGL3* empty vector, Promega) were transfected into 10^6^ hESCs using the Neon transfection system (Invitrogen). The *pRL-TK* plasmid (Promega), which expresses *Renilla*, was co-transfected as an internal control (75 ng per well). At 48 h post transfection, luciferase assays were performed using the dual-luciferase assay system (Promega). Activities of both firefly and *Renilla* luciferases were measured using a Glomax microplate luminometer (Promega). Relative luciferase was calculated by normalizing firefly luciferase activity (reporter) to *Renilla* luciferase activity (internal control).

### Virus production and infection

To produce SIV-derived lentivectors, 293T cells were transfected with a mixture of DNA containing 7.5 μg of a *pGRev* plasmid encoding for the vesicular stomatitis virus glycoprotein envelope; 4 μg of a *pSIV3*^*+*^ plasmid encoding for the gag, pol, tat and rev proteins; and 11.5 μg of the vector plasmid (*pGAE-STAT3-ER*^*T2*^) using the calcium phosphate precipitation technique. Culture supernatant was collected after 2 days, centrifuged to remove cells and debris, and directly used to infect hESCs. Before infection, undifferentiated OSCAR (P16) and H9 (P28) hESCs were manually collected and treated with 0.05% trypsin-EDTA for 8 min at 37 °C until the clumps were dissociated into single cells. After centrifugation, the cells were resuspended at the density of 6 × 10^4^ cells per ml in conventional ES medium containing GAE-STAT3-ER^T2^ lentivirus in the presence of 10 μg ml^−1^ polybrene (Sigma) and 10 μM Rock inhibitor Y-27632 (Calbiochem). The cells were incubated for 5 h at 37 °C in 5% O_2_ before being replated at a clonal density into 96-well plates containing MEF in medium supplemented with 20% KOSR and 4 ng ml^−1^ of FGF2.

### *In-vitro* differentiation and generation of teratomas

For embryoid body (EB) formation, hESCs were collected after manual splitting into small clumps (TL cells), or by dissociation using 0.05% trypsin-EDTA (TL2i cells). The cells were then suspended in drops in ES basal medium (around 200–500 cells per 30 μl per drop) for 2 days. Two-day-old EBs were transferred to a DMEM (Gibco) suspension medium supplemented with 10% fetal bovine serum (Gibco), 1% penicillin–streptomycin–glutamine (Gibco), 1% NEAA and 0.1 mM β-mercaptoethanol. At day 8, the EBs were randomly chosen and plated onto 0.5% gelatin-treated coverslips in four-well plates. The cell culture medium was replaced once a week until the cells were fixed with 4% paraformaldehyde (PFA, Sigma) for immunocytochemistry on day 22. For the generation of teratomas, cells at a density of 0.5–1.0 × 10^6^ were injected into the testis of 8-week-old SCID mice (CB17/SCID, Charles River; *n*=1–3). After 4–10 weeks, the mice were euthanized and teratomas were surgically dissected and fixed in 4% PFA for cryosectioning. The 20-μm-thick sections were stained with haematoxylin and eosin.

### Karyotype analysis

To arrest the cells at metaphase, subconfluent hESCs were incubated with 0.1 μg ml^−1^ KaryoMax colcemid (Gibco) in knockout DMEM at 37 °C in 5% O_2_ for 90–135 min. Cells were washed with PBS and digested with 0.05% trypsin-EDTA at 37 °C for 10 min. Cells were resuspended in DMEM medium supplemented with 10% fetal bovine serum and centrifuged for 8 min at 1,000 r.p.m. The supernatant was removed and the cell pellets were resuspended in 9 ml of 0.075 M KCl and incubated at 37 °C for 20 min. The cells were then fixed with freshly prepared methanol/acetic acid (3:1, v/v). G-band karyotyping was performed by Cell Guidance Systems (UK). Twenty metaphases from each cell sample were randomly selected and analysed.

### Kinase inhibition assay

Undifferentiated ES cell colonies were manually split into small clumps and evenly distributed onto 35-mm dishes or 24-well plates. After 24 h, the cells were treated with JAK2 inhibitor III (10 μM, Calbiochem 573098), FGF receptor-specific inhibitor SU5402 (25 μM, unless otherwise indicated, Calbiochem 572630), or activin receptor-like kinase inhibitor SB431542 (10 μM, unless otherwise indicated, Sigma S4317) for 3–5 days, before being harvested for qPCR or fixed for AP assay. Dimethyl sulfoxide, the inhibitors' solvent, was added alone to the cells as control.

### Immunofluorescence and immunoblotting

Cells were fixed with 4% PFA in PBS for 20–30 min and permeabilized in 0.4% Triton X-100 (Sigma) for 10 min. Nonspecific binding sites were blocked with 10% goat serum for 30 min at room temperature. Cells were then incubated overnight at 4 °C or 1 h at room temperature (RT) with primary antibodies (Supplementary Table S4). After three rinses in PBS, cells were exposed to goat anti-mouse, anti-rabbit immunoglobulin G, or immunoglobulin M conjugated either to fluorescein isothiocyanate, Alexa-488, -555, or -647 (dilution 1:1,000) (Invitrogen) for 1 h at room temperature, followed by nuclear staining with 5 μg ml^−1^ Hoechst 33258 for 8 min. After three rinses in PBS, coverslips were mounted on slides. The cells on coverslips were examined using a confocal laser scanning system (Confocal SP5, Leica Microsystems).

For immunoblotting, frozen cell pellets were lysed in RIPA buffer complemented with protease and phosphatase inhibitors. Protein lysates were then cleared by centrifugation (14,000 r.p.m. for 30 min). After SDS–PAGE and electroblotting on polyvinylidene difluoride, the membranes were incubated with specific primary antibodies (Supplementary Table 4). Blots were incubated with horseradish peroxidase-coupled anti-mouse immunoglobulin G (Jackson ImmunoResearch) and developed with Clarity Western ECL Substrate (BIO-RAD).

For 5mC and 5hmC staining, cells were briefly washed in PBS and fixed in 4% PFA for 10 min at RT. The cells were then permeabilized by using PBS, 1% BSA, 0.5% Triton X-100 for 30 min and DNA was denatured in 2 N HCl for 15 min at RT, followed by a neutralization using 1 M Tris-HCl buffer (pH 8, 10 min at RT). After extensive washes, slides were blocked with PBS, 1% BSA, 0.1% Triton X-100 for 30 min and then incubated with 5hmC (Active Motif 39791, 1:400) and 5mC (Diagenode 081100, 1:4,000) at 4 °C overnight. The slides were washed three times in PBS, 1% BSA, 0.1% Triton X-100 and incubated with Alexa fluorophore-conjugated secondary antibodies (Invitrogen) for 1 h at RT in the dark and washed three times in PBS, 1% BSA, 0.1% Triton X-100, and twice in PBS. Finally, the slides were incubated with DAPI (4,6-diamidino-2-phenylindole; Life Technologies) in PBS for 5 min in the dark, washed three times in PBS, mounted with Mounting Medium (Sigma) and imaged by using a Leica SP5 confocal microscope.

AP activity was revealed using the AP substrate kit (Ref. 86R; Sigma-Aldrich), following the manufacturer's instructions.

### RNA FISH and microscopy

Alexa-488-labelled probes were generated by nick translation for *XIST* (a 10-kb fragment corresponding to *XIST* exon 1, a gift from E. Heard) and *ATRX* (RP11–42M11, BACPAC). Alexa-594-labelled probes were generated by nick translation for *XACT* (RP11–35D3, BACPAC). All probes generated from BACs were precipitated with human Cot-1 DNA (Life Technologies) and the *XIST*-probe with Salmon Sperm DNA (Life Technologies), resuspended in formamide and hybridization buffer, and denatured for 7 min at 75 °C. Cot-1 precipitated probes are additionally pre-incubated 15 min at 37 °C. Cells were grown on coverslips and coverslips were dehydrated in 90% and 100% ethanol and incubated overnight with probe at 37 °C. After three 50% formaldehyde/2 × SSC washes and three 2 × SSC washes at 42 °C for 4 min, coverslips were mounted in Vectashield plus DAPI. All images were taken on a fluorescence microscope Axioplan 2 Imaging (Zeiss) with a cooled Coolsnap camera (Roper Scientifics) controlled by the Metamorph 7.04 software (Roper Scientific) using a Plan-neofluar × 100 oil objective (numerical aperture 1.3, Zeiss). Optical Z-sections were collected at 0.2 μm steps through each nucleus at different wavelengths depending on the probes used (DAPI (360 and 470 nm), fluorescein isothiocyanate (470 and 525 nm) and Texas Red (596 and 612 nm)); ∼25 optical sections per nucleus were collected. Stacks were processed using ImageJ 1.46, and throughout the manuscript the three-dimensional FISH experiments are represented as a two-dimensional projection of the stacks (maximum projection).

### Real-time PCR

RNA was extracted using RNAeasy kits with on-column DNAse digestion and reverse transcription carried out with MuMLV-RT (Promega), according to the manufacturer's recommendations. Oligonucleotide sequences are listed in Supplementary Table 5. qPCR was performed using the LightCycler 1.5 system and the LightCycler Fast Start DNA Master SYBR Green I kit (Roche Applied Science), according to the manufacturer's instructions. All normalizations were carried out using the *GAPDH* gene.

### Microarrays and bioinformatics

Each cell line was analysed in triplicate. Total RNAs from hESCs were prepared using a Qiagen column kit (Qiagen) and treated with DNAse (5 U per 100 mg RNA, Sigma). Complementary RNA was prepared using the IVT Express (200–100 ng) kit (Affymetrix), according to the manufacturer's instructions, starting from 100 ng total RNA. Following fragmentation, complementary RNA was hybridized for 16 h at 45 °C on the GeneChip Human U219 array. GeneChips were washed and stained in the Affymetrix Fluidics Station 450. GeneChips were scanned using the Affymetrix GeneChip Scanner 3000 7G. The image data were analysed with the GeneChip Operating Software, using Affymetrix default analysis settings. Arrays, after passing the quality control, have been commonly normalized by using the log-scale robust multi-array analysis^52^. After outlier removal using the Nalimov test at *P*<0.001, a parametric analysis of variance (ANOVA; F-test) was applied, to determine global expression differences among various conditions. Hierarchical clustering of expression values of probe sets differentially expressed in the ANOVA was performed by using the function heatmap2 of the gplots R package, with default hierarchical clustering method (rows by Pearson correlation as distance method and columns by Spearman correlation as distance method).

Cross-species gene expression analysis and transcriptome meta-analysis of naive human PSCs were conducted on our human data set and previously described mESCs and EpiSCs, and naive and primed hESC gene expression data sets (GSE23402, GSE15603, GSE7866, GSE29397, GSE43421 and GSE43398). We used the VirtualArray R/Bioconductor package^53^ to combine and normalize the different data sets, collapse by median probes targeting the same genes and identify genes present in all data sets, using gene symbols as common identifiers. The newly generated expression sets were then subjected to batch effect removal using empirical Bayes methods. ANOVA (F-test) was applied to obtain global expression differences between the different conditions. We used R and the R package FactoMine to perform PCA on the differentially expressed genes in the ANOVA. GO analysis was performed using the online tool Gorilla (http://cbl-gorilla.cs.technion.ac.il/).

### Chromatin immunoprecipitation

ChIPs for H3K4me3 and H3K27me3 histone marks were performed on F and TL2i H9S3 and OS3 hESCs, using previously described protocols^31^. In brief, 10^7^ cells were cross-linked with 1% formaldehyde for 10 min at RT and formaldehyde was inactivated by the addition of 125 mM glycine. Chromatin from F and TL2i hESCs was sonicated to a length of ∼250–500 bp and subsequently immunoprecipitated using H3K4me3 and H3K27me3 antibodies (Diagenode: pAb-003-050 and pAb-195-050). Samples were sequenced using the Illumina Genome Analyzer IIx platform. ChIP sequencing reads were aligned using the Bowtie software to GRCh build 37 (hg19) of the human genome. The SICER programme (Statistical approach for the Identification of ChIP-Enriched Regions) was used to detect peaks of ChIP enrichment and run with the default settings, using a fragment size of 150 and a gap size of 600. Redundant reads that could result from the overamplification of ChIP DNA were removed and peak enrichment was calculated relative to the genome background. A threshold of *P*=10^−10^ was used to call significant peaks. An input control sample was also included to eliminate non-random enrichment. Binding profiles were generated using IGV version 2.3.

### Single-cell gene expression qPCR

Cells were dissociated using Accutase, filtered and checked for single cells suspension quality. About 600 single cells were introduced in the cell input well of the C1 Array IFC (10–17 μm). Single cells captured on the C1 Array were harvested after loading, live-dead stained with a Live/dead Cell viability/Cytotoxicity kit (Invitrogen L3224), imaged and subjected to reverse transcription and specific-target-amplification, using components from the Cells-to-Ct kit (Ambion 4458237), C1 Single-Cell Auto Prep Modules Kit (Fluidigm 100–5319) and pooled primers (500 nM). These preamplified products were diluted 100-fold before analysis with Universal PCR TaqMan Master Mix (Applied) coupled with a DNA Binding Dye Sample Loading Reagent (Fluidigm) and Evagreen (Biotium 31,000) in 96.96 Dynamic Arrays on a BioMark System (Fluidigm), following the manufacturer's instructions. After loading, the C1 Array IFC was transferred to the BioMark and PCR was performed using the following thermal protocol: thermal mix of 50 °C 2 min, 70 °C 30 min, 25 °C 10 min; hot start at 50 °C 2 min, 95 °C 10 min; PCR cycle of 35 cycles of (95 °C 15 s, 60 °C 60 s); and melting analysis. Bulk cell suspension samples, as well as positive and negative controls, were prepared in parallel. All oligonucleotide sequences are shown in Supplementary Table 5.

### Single-cell data processing and visualization

Each assay was performed in replicates. For subsequent analysis, we selected cells and genes as follows: for each gene, inconsistent melting curve, ‘failed' quality control readings and inconsistent expression between replicates was filtered out. Similarly, each cell with failed or inconsistent detection of control genes (*TBP* and *GAPDH*) was removed from the analysis. Expression values were deduced from Ct and ΔΔCt values calculated from the system's software (BioMark Real-time PCR Analysis, Fluidigm) using the cellular pool as a sample reference and *GAPDH* or *TBP* as reference genes. Data were visualized first by using the R package ‘SINGuLAR Analysis Toolset' developed by Fluidigm. Multidimensional scaling and clustering were conducted using the R software on Euclidian distances or correlation. Heatmaps were generated using the heatmap.2 function of the R package ‘gplots'. To identify differentially expressed genes, we performed a one-way ANOVA, to test for differences in gene expressions among identified cellular subgroups.

### Reduced representation bisulfite sequencing

We prepared RRBS libraries according to a published protocol^54^, with modifications. We digested 200 ng of genomic DNA for 5 h at 37 °C with MspI (Fermentas), performed end repair and A-tailing for 40 min at 37 °C using a Klenow fragment (Fermentas) and ligated the fragments to methylated paired-end adapters overnight at 16 °C using a T4 DNA ligase (Fermentas). We then purified the short fragments (150–400 base pairs, including the size of the adapter) by electrophoresis on a 3% agarose 0.5 × TBE gel with the MinElute kit (Qiagen) and performed two rounds of bisulfite conversion with the EpiTect kit (Qiagen). We generated RRBS libraries by PCR amplification with PfUTurbo Cx hotstart DNA polymerase (Agilent) and indexed Illumina primers using the following PCR conditions: 95 °C for 2 min, 12 cycles (95 °C for 30 s, 65 °C for 30 s, 72 °C for 45 s) and 72 °C for 7 min. The libraries were purified with AMPure magnetic beads (Beckman Coulter) and paired end sequenced (2 × 75 bp) in multiplex on an Illumina HiSeq2000, to generate ∼35 million pairs of reads per sample. We performed quality controls on sequencing reads with FastQC, trimmed sequencing reads with Trim Galore to remove adapter sequences and low-quality ends with a Phred score below 20, and aligned to the traces of the human genome using BSMAP. We extracted methylation scores as the ratio of the number of Cs over the total number of Cs and Ts, and only retained CpGs sequenced at least 8 × (∼3 million CpGs per sample). The bisulfite conversion efficiency estimated by calculating the C to T conversion at non-CpG sites was >99%. The comparative analysis of methylation was performed on the set of CpGs with at least 8 × coverage in both data sets (total *n*=2,879,485 CpGs). Data processing was performed with the R software.

## Additional information

**Accession codes:** Microarray, ChIP sequencing and RRBS data are available in the Gene Expression Omnibus database (http://www.ncbi.nlm.nih.gov/geo/) under the accession numbers GSE55708 (microarray data), GSE61224 (ChIP sequencing data) and GSE61224 (RRBS data).

**How to cite this article:** Chen, H. *et al.* Reinforcement of STAT3 activity reprogrammes human embryonic stem cells to naive-like pluripotency. *Nat. Commun.* 6:7095 doi: 10.1038/ncomms8095 (2015).

## Supplementary Material

Supplementary Information Supplementary Figures 1-13 and Supplementary Tables 1-3
