# Supplementary material for: Reinforcement of STAT3 activity reprogrammes human embryonic stem cells to naive-like pluripotency
Source: Nat Commun. 2015 May 13;6:7095. doi: 10.1038/ncomms8095 (PMC4479042; doi:10.1038/ncomms8095)
Supplement: Supplementary Information — Supplementary Figures 1-13 and Supplementary Tables 1-3 [file ncomms8095-s1.pdf]

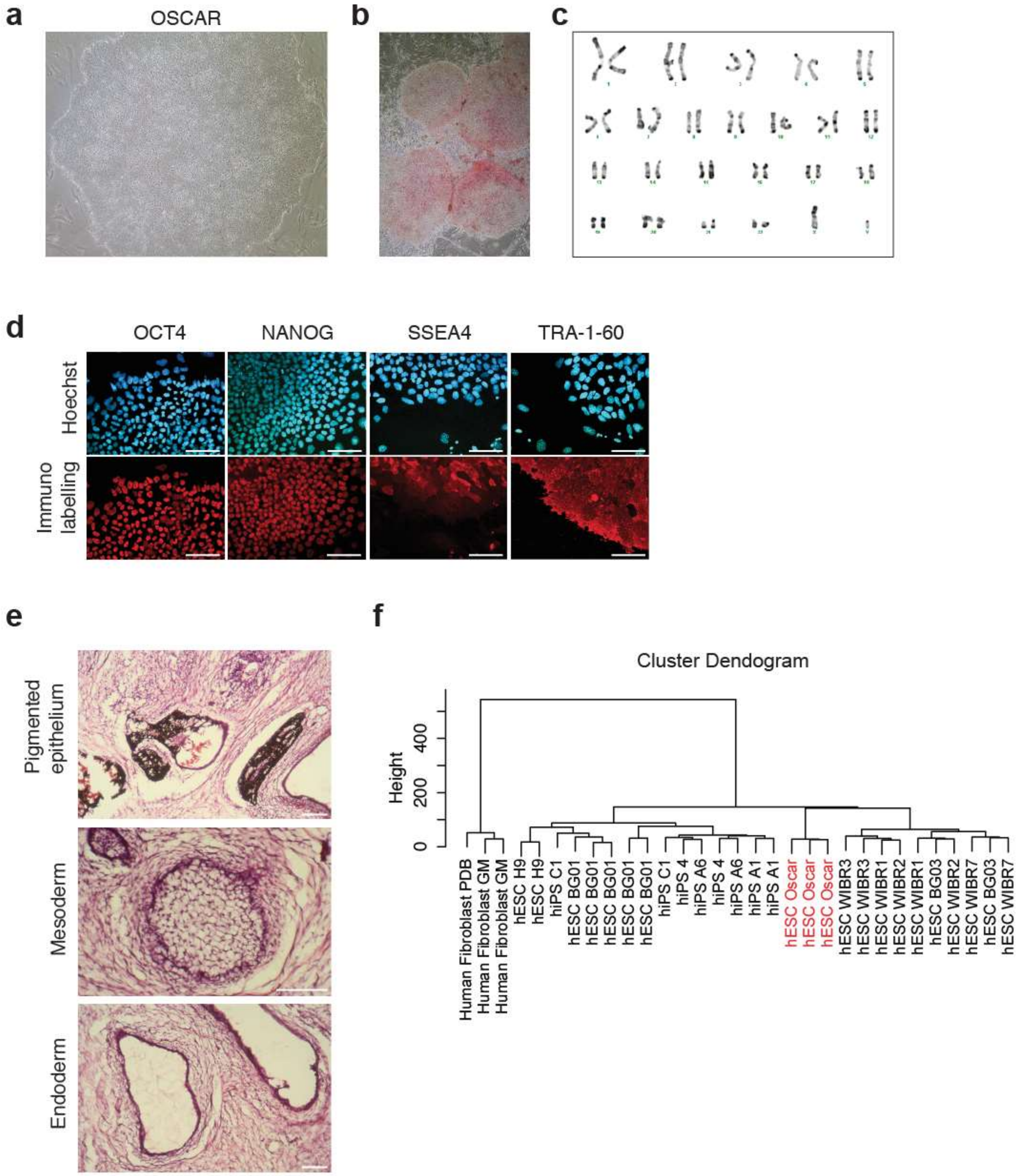


**Supplementary File 1: Establishment and characterization of the OSCAR hESC line.**

METHODS: Sixty-eight frozen-thawed donated human embryos produced by IVF were cultured to the blastocyst stage in 199 medium (Sigma). The inner cell masses (ICMs) were isolated by immunosurgery, with a rabbit antiserum to human cells with guinea pig complement (Sigma), and plated on mitomycin C-treated mouse embryonic fibroblasts (MEFs). The culture medium consisted of 80% Knockout Dulbecco's modified Eagle's medium (DMEM, high glucose formulation; Gibco) supplemented with 20% Knock-Out Serum Replacement (KO-SR), 1 mM of glutamine, 0.1 mM of β-mercaptoethanol (Sigma), 1% nonessential amino acid (Gibco), 1 mM of sodium pyruvate (Gibco), and 8 ng/mL of FGF2 (Millipore). After 7 days, the ICM-derived outgrowths was dissociated into clumps by mechanical

dissociation with a micropipette, and re-plated on growth-inactivated MEF in fresh medium. Individual colonies with a uniform undifferentiated morphology were individually selected by micropipette, mechanically dissociated into clumps, and re-plated. Once established, the ES cells were passaged as described in the next section.

RESULTS: OSCAR cells were routinely passaged by mechanical dissociation and propagated onto growth-inactivated MEFs. They expressed the cardinal markers of hESCs, exhibited a normal 46XY chromosomal complement and could differentiate into derivatives of the three germ layers in experimental teratomas. The transcriptomes of OSCAR cells was compared with those of 7 other hESC lines (BG01, BG03, WIBR1, WIBR2, WIBR3, WIBR7, and H9), 2 hiPSC lines, and 3 fibroblast lines (GSE23402) (Guenther et al., 2010). Hierarchical clustering analysis showed that the OSCAR hESCs were transcriptionnaly similar to all the other hESC and hiPSC lines, further confirming their pluripotent stem cell identity. (a) Phase contrast microphotograph of OSCAR colony. (b) Detection of alkaline phosphatase activity in OSCAR cells. (c) G-banded karyotype of the OSCAR cell line at P34. (d) Immunofluorescence labeling of the OSCAR hESCs with antibodies to OCT4, NANOG, SSEA-4, and TRA-1-60. (e) Teratoma sections after hematoxylin and eosin staining. Scale bar, 100 µm. (f) Ward hierarchical clustering of transcriptome data.

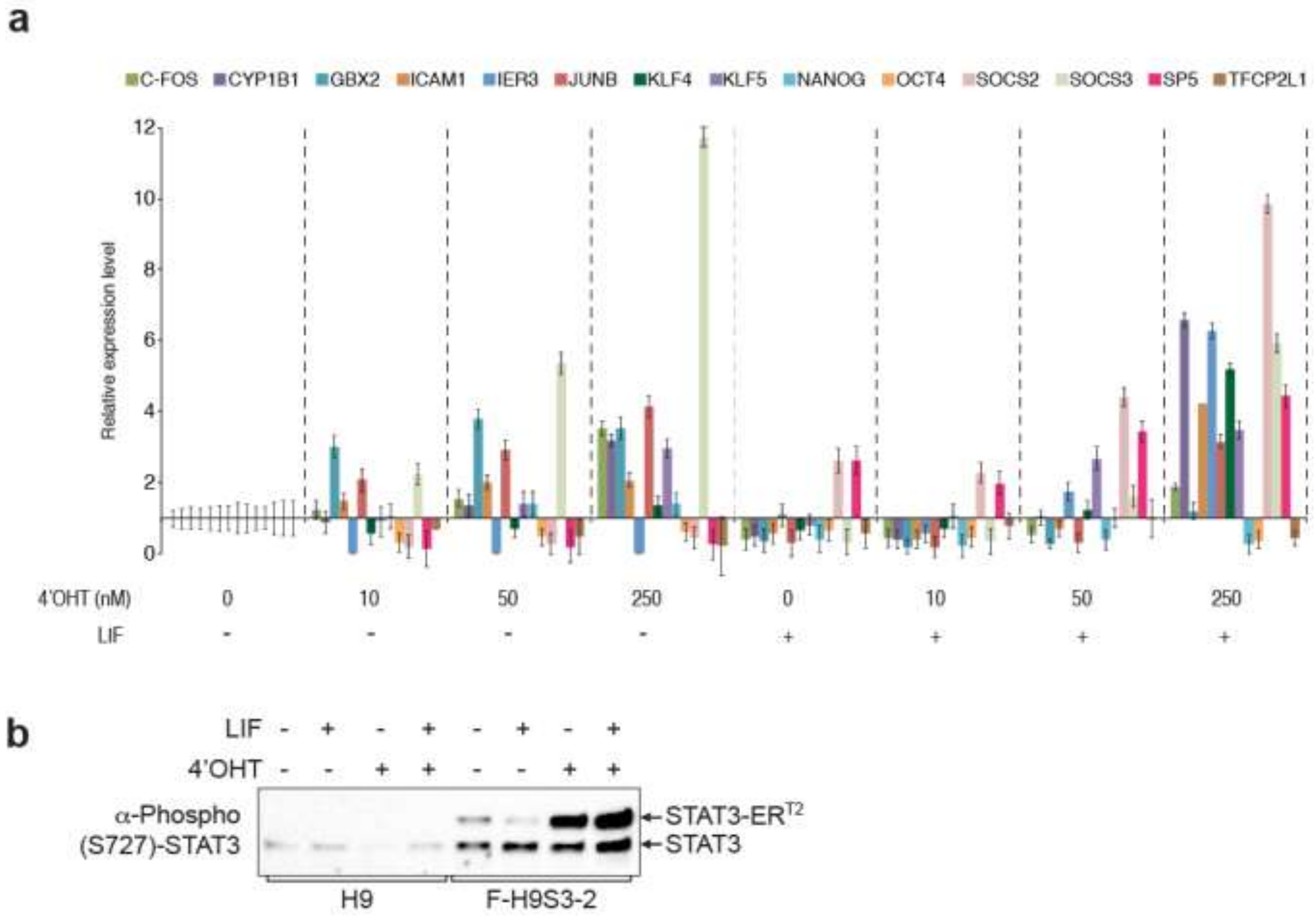


**Supplementary File 2:**

**(a) Activation of STAT3 target genes in F-OS3-10 hESCs after stimulation with LIF and 4'OHT**. We examined the expression of 12 STAT3 target genes, after stimulation for 4 days with LIF, 4'OHT, or both. The histogram represents the mRNA level (ΔCt) of the STAT3 target genes, measured by quantitative RT-PCR in F-OS3-10 cells after consecutive stimulation with 10,000 U/ml LIF, 0-250 nM 4'OHT, or with both for 4 days. All ΔCt were normalized to GAPDH (ΔCt = 1; n=3, average ± s. d.). Stimulation with LIF induced a moderate activation of SOCS2 and SP5. On the other hand, 4'OHT induced a much stronger activation of C-FOS, CYP1B1, GBX2, JUNB, IER3, KLF5, and SOCS3 in a dose-dependent manner. Moreover, when 4'OHT stimulation was performed in the presence of LIF, KLF4, SOCS2, and SP5 were also activated. These data suggest that 4'OHT was more efficient in activating the expression of STAT3 target genes compared with LIF. In addition, some target genes were only activated on stimulation with both LIF and 4'OHT, suggesting that the two factors act in synergy to regulate the transcription of STAT3 target genes.

**(b) Response of STAT3 and STAT3-ERT2 to stimulation with LIF and 4'OHT in F-H9S3-2 cells.** Western blot analysis of phospho-(Ser727)-STAT3 expression in H9 and F-H9S3-2 cells after stimulation for 1 h with 10,000 U/mL LIF, 250 nM 4'OHT, or both. We could detect phosphorylated-(Ser727)-STAT3 at an elevated level in F-H9S3 cells, regardless of LIF or 4'OHT. This latter observation is in line with a recent report that primed pluripotency is associated with constitutive phosphorylation of STAT3 on serine 727 (Huang et al., 2014). Of note, phosphorylated-(Ser727)-STAT3-ERT2 level was dramatically increased by 4'OHT in the F-H9S3 cells, regardless of LIF. The role of phosphor-Ser727 on the activity of STAT3-ERT2 is not known.

Huang, G., Yan, H., Ye, S., Tong, C. & Ying, Q. L. STAT3 Phosphorylation at Tyrosine 705 and Serine 727 Differentially Regulates Mouse ESC Fates. *Stem Cells* **32**, 1149-1160, doi:10.1002/stem.1609 (2014).

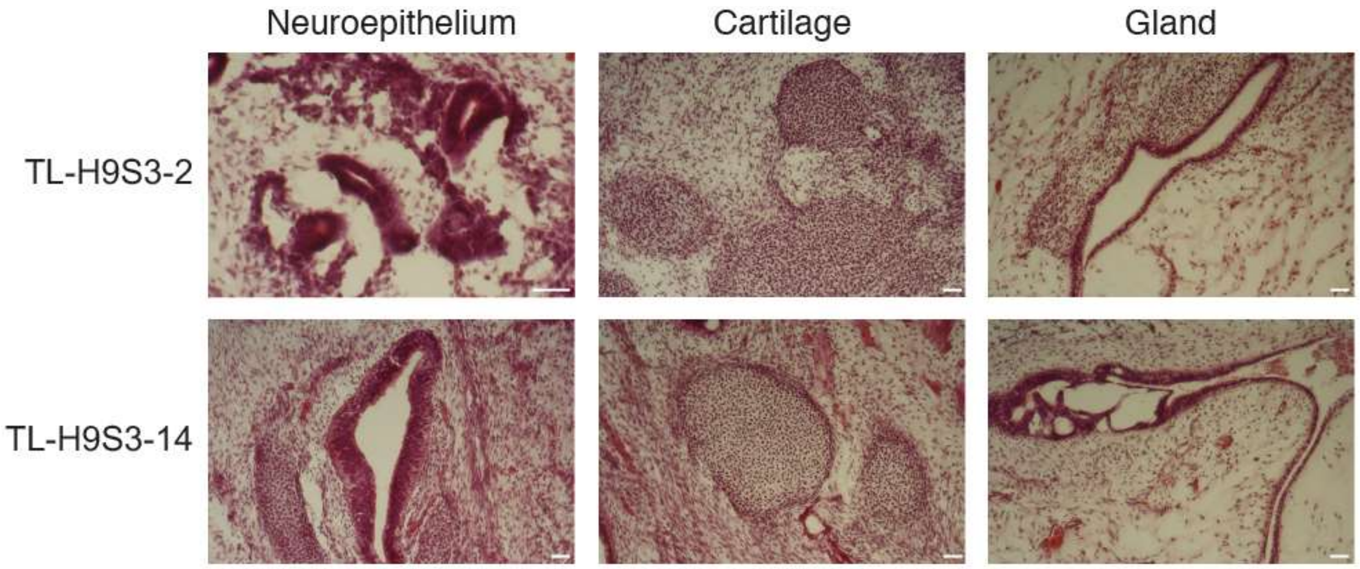


**Supplementary File 3: Differentiation of TL-H9S3-2 and TL-H9S3-14 cells in vivo by teratoma formation.**

H&E staining. Scale bar, 50 μm.

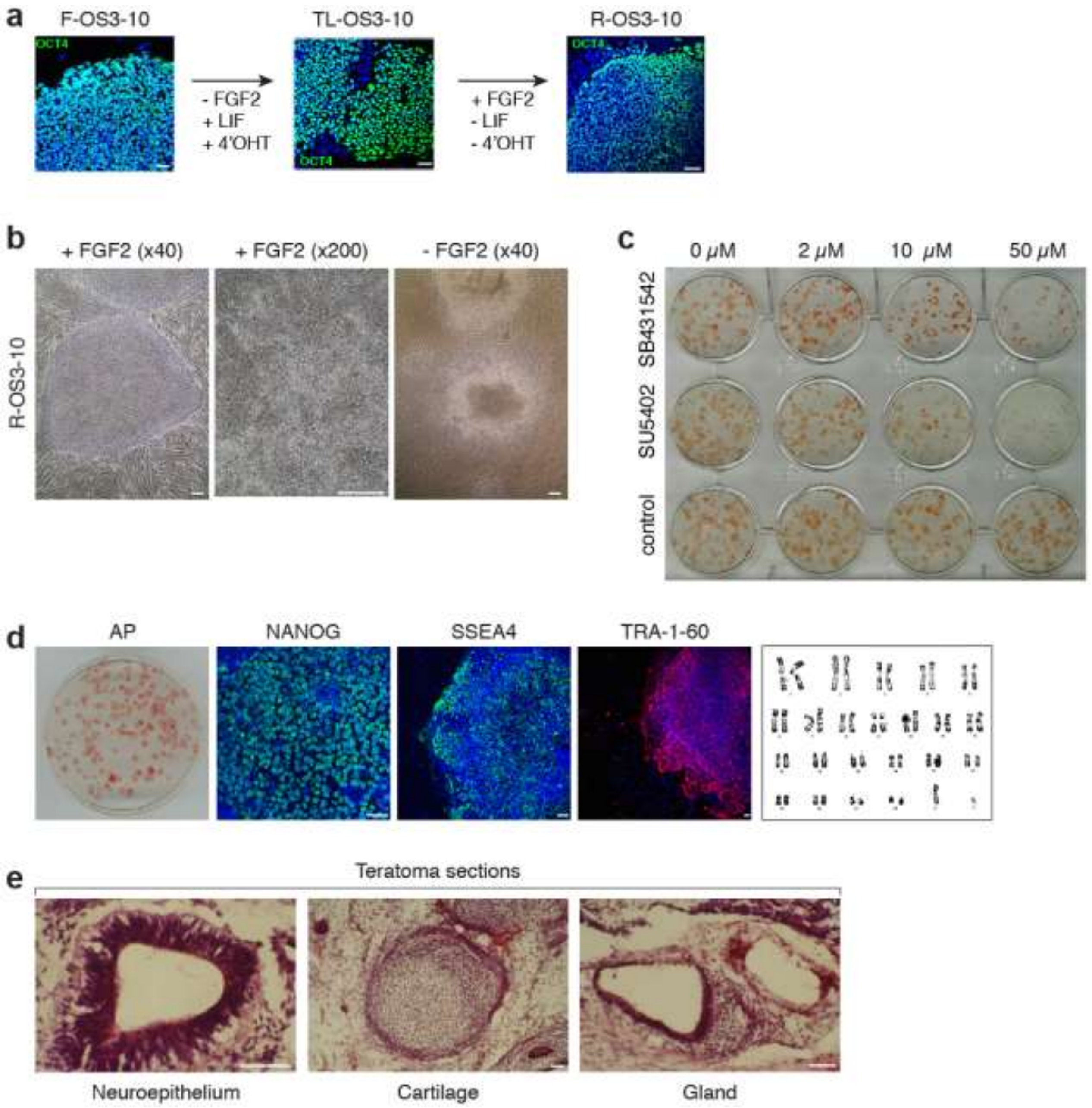


**Supplementary File 4: Reversion of TL-OS3-10 cells to conventional FGF2 dependent human ES cells (R-OS3-10 cells).**

To assess whether the TL cells could be reverted to a FGF2-dependent state, the TL-OS3-10 cells were transferred back to a culture medium lacking LIF and 4'OHT and supplemented with 5 ng/mL FGF2. The resulting cells were designated as Reverted R-OS3-10 cells. Both the withdrawal of FGF2 and treatment with the FGFR inhibitor SU5402 or with the SMAD inhibitor SB431542 induced differentiation of the R-OS3-10 cells, demonstrating FGF2 and activin dependency. The R-OS3-10 cells expressed the alkaline phosphatase (AP), OCT4, NANOG, SSEA4, and TRA-1-60, and could develop teratomas that contained derivatives of the three germ layers. These results suggest that OS3-10 hESCs can self-renew in two interchangeable states of pluripotency, one that exploits FGF2-signalling, and the other one LIF/STAT3-signalling. (**a**) Schematic representation of the conversion of F-OS3-10 cells into TL-OS3-10 cells, then of TL-OS3-10 cells into R-OS3-10 cells. Pictures represent immunolabelling of the indicated cell types with OCT4 antibody. (**b**) Microphotographs of R-OS3-10 cells (+/- FGF2). Scale bar, 50 µm. (**c**) Colony-forming assay of R-OS3-10 cells with SU5402 and SB431542 inhibitors, demonstrating dependency on FGF and activin receptor activity, respectively. (**d**) Alkaline phosphatase activity, and immunofluorescence labeling of NANOG, SSEA4, and TRA-1-60, and normal G-band karyotyping in R-OS3-10 (P10). (**e**) Neuroepithelium (ectoderm), cartilage (mesoderm), and gland (endoderm) differentiation in teratoma from R-OS3-10 cells after H&E staining. Scale bar, 50 µm.

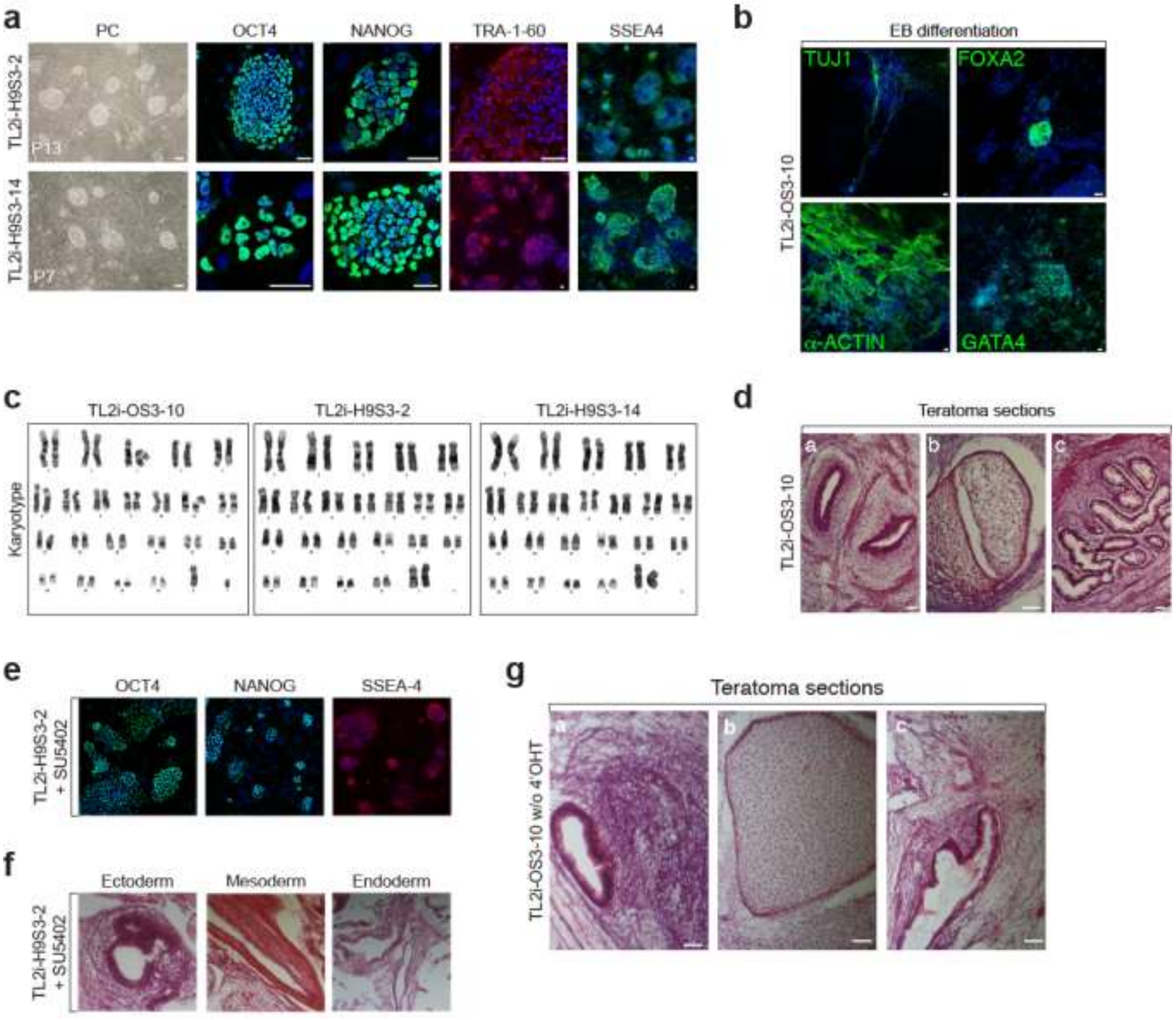


**Supplementary File 5: Characterization of TL2i cells.**

(**a**) Phase contrast microphotograph and immunofluorescence labeling of TL2i-H9S3-2 and TL2i-H9S3-14 cells with antibodies to OCT4, NANOG, TRA-1-60, and SSEA-4. Scale bar, 50 µm. (**b**) Immunofluorescence labeling of TL2i-OS3-10 cells with antibodies to TUJ1 (neurons), FOXA2 and GATA4 (endoderm), and α-ACTIN (heart) after differentiation induced by formation of embryoid bodies. (**c**) G-banding karyotype of the TL2i-OS3-10 cells at P10, and TL2i-H9S3-2 and TL2i-H9S3-14 at P6. (**d**) Teratoma formation with TL2i-OS3-10 cells. H&E staining shows neurectodermal (a), cartilage (b), and gut-like structure (c). (**e**) Immunofluorescence labeling of TL2i-H9S3-2 cells cultured in medium supplemented with SU5402, with antibodies to OCT4, NANOG, and SSEA-4. Scale bar, 50 µm. (**f**) Teratoma formation with TL2i-H9S3-2 cells after propagation for 24 days in the presence of FGFR inhibitor (SU5402 at 10 µM). H&E staining shows neurectoderm, muscle, and gut-like structures. (**g**) Teratoma formation with TL2i-OS3-10 cells after propagation for 6 passages in the absence of 4'OHT. H&E staining shows neurectodermal (a), cartilage (b), and gut-like structure (c).

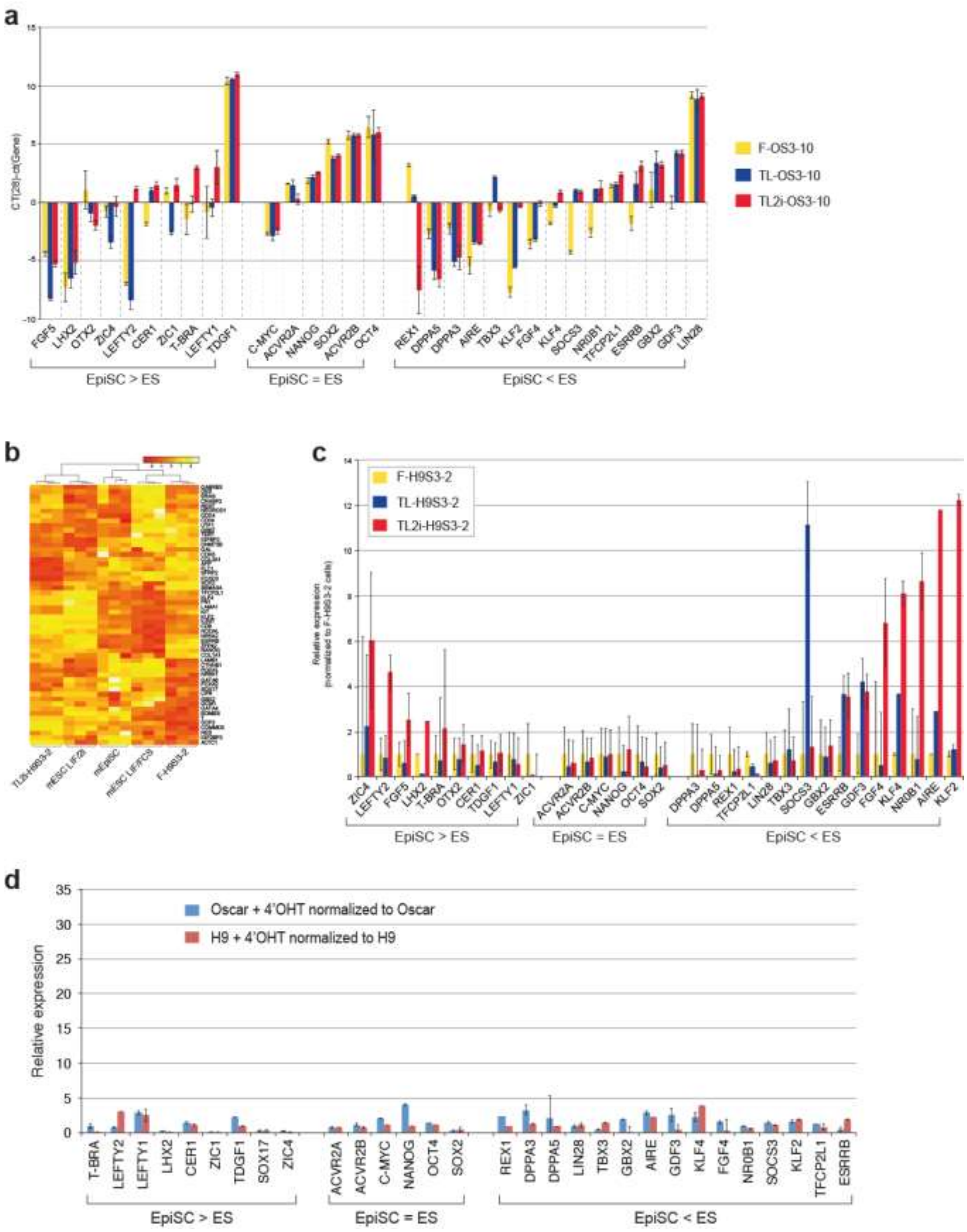


**Supplementary File 6: Analysis of naïve and primed pluripotency markers in F, TL and TL2i cells.**

(**a**) Histogram of expression level distributions across F, TL and TL2i cells for pluripotency and lineage marker genes. Each cell type is shown in a separate color. A background of Ct = 28 was used to calculate the absolute expression level. EpiSC>ES indicates genes that are overexpressed in mEpiSCs as compared with mESCs; EpiSC=ES indicates genes that are equally expressed in mEpiSCs and mESCs; EpiSC<ES

indicates genes that are overexpressed in mESCs as compared with mEpiSCs. (**b**) Non-supervised cross-species comparison of the transcriptome of mESCs cultivated in conventional medium (FCS + LIF) and 2i/LIF medium, EpiSCs, F-H9S3-2 and TL2i-H9S3-2 cells. (**c**) Histogram representation of the mRNA level (ΔCt) of pluripotency and lineage marker genes in F-H9S3-2, TL-H9S3-2, and TL2i-H9S3-2, after normalization to *GAPDH* (ΔCt = 1). (**d**) Expression of pluripotency markers (naive and primed), after stimulation of hESCs with 4'OHT. The histogram represents the mRNA level (ΔCt) of the genes measured by quantitative RT-PCR in Oscar and H9 cells after stimulation with 250 nM 4'OHT for 21 days, and after normalization to the mRNA level measured after stimulation with the vehicle alone. All ΔCt were normalized to *GAPDH* (ΔCt = 1; n=3, average ± s. d.) prior to normalization to control cells. Most genes analyzed showed light to moderate activation after stimulation with 4'OHT (< 4-fold). However, with most of them, levels remained well below those measured in TL and TL2i cells. This indicates that activation of naïve markers in TL2i-OS3 and TL2i-H9 cells results from cell reprogramming by STAT3-$ER^{T2}$ and LIF, and not from a direct effect of 4'OHT on the transcriptional regulation of naïve pluripotency genes.

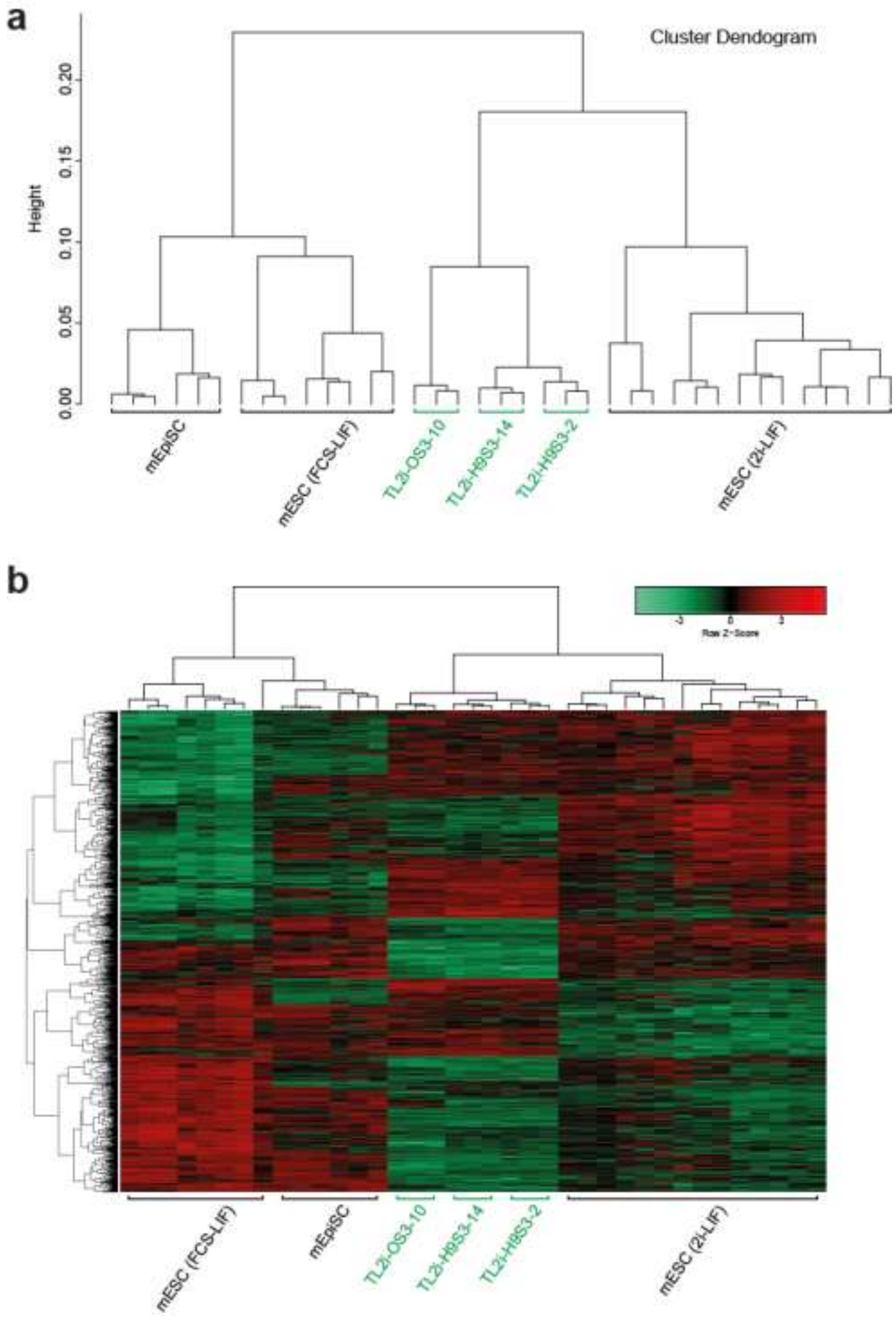


**Supplementary File 7: Transcriptome reconfiguration of TL2i cells.** TL2i cells cluster with mESCs propagated in 2i+LIF conditions.

(**a**) Correlation clustering of mEpiSC, mESC (FCS-LIF), mESC (2i-LIF) and TL2i cells transcriptome data. (**b**) Hierarchical clustering and heatmap of transcriptome data (mean values/cell category most differentially expressed 1000 probe sets) using Pearson correlation coefficient as a measure of distance between rows and Spearman correlation coefficient as a measure of distance between columns.

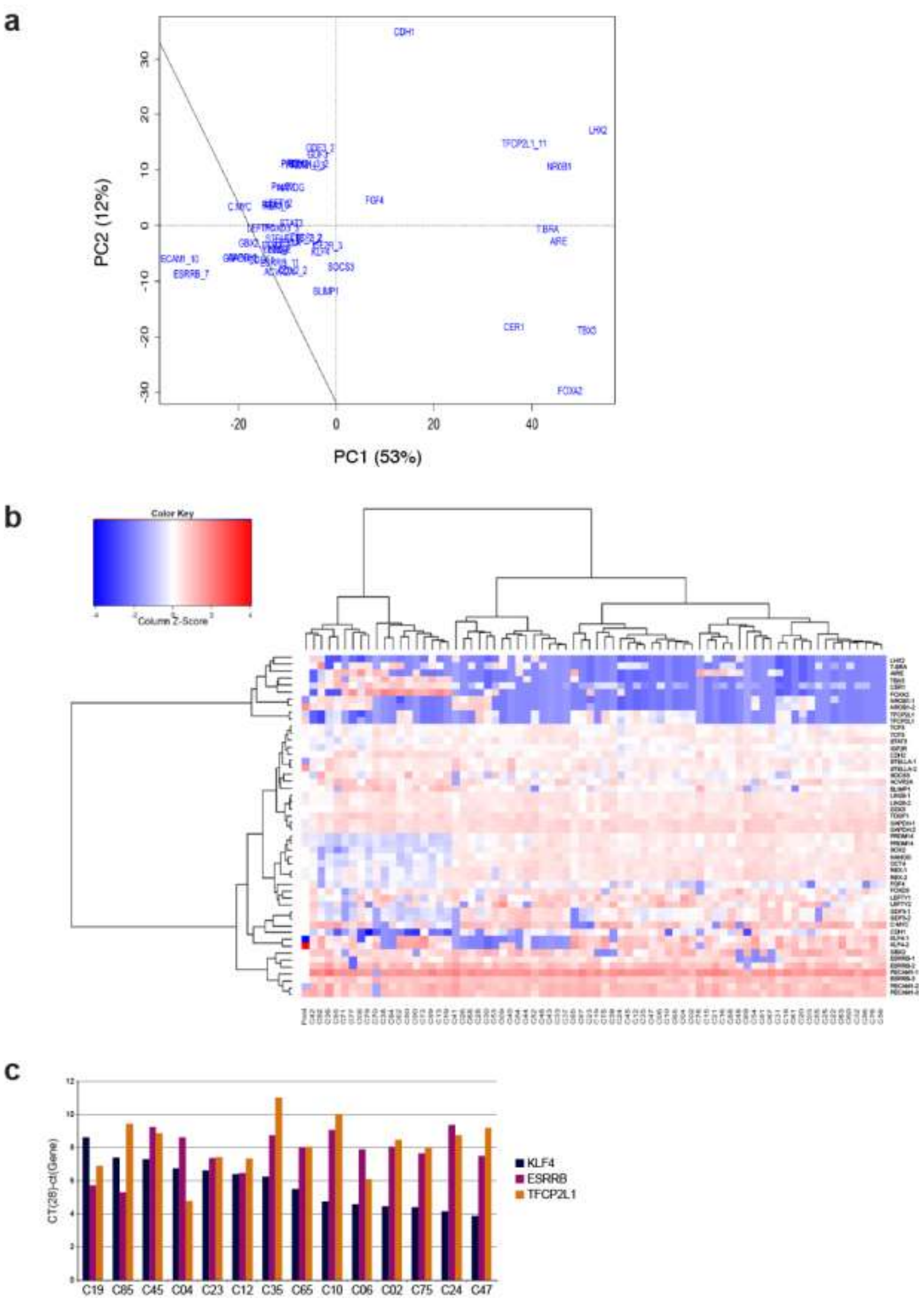


**Supplementary File 8: Single-cell PCR analysis of the TL2i-H9S3-2 population.**

(**a**) Graphical representation of the first principal component of PCA for the 35 genes analyzed. (**b**) Representative Heatmap of normalized Fluidigm data for TL2i-H9S3-2 cells. Each column corresponds

to a single cell while rows correspond to genes. Blue and red gradations highlight, respectively, downregulated and upregulated genes compared to a random pool of TL2i-H9S3-2 cells. (**c**) Histogram of expression level distribution in 15 TL2i cells obtained by single-cell qPCR analysis for *KLF4*, *ESRRB* and *TFCP2L1*. Each gene is shown in a separate color. A background of Ct = 28 was used to calculate absolute expression levels.

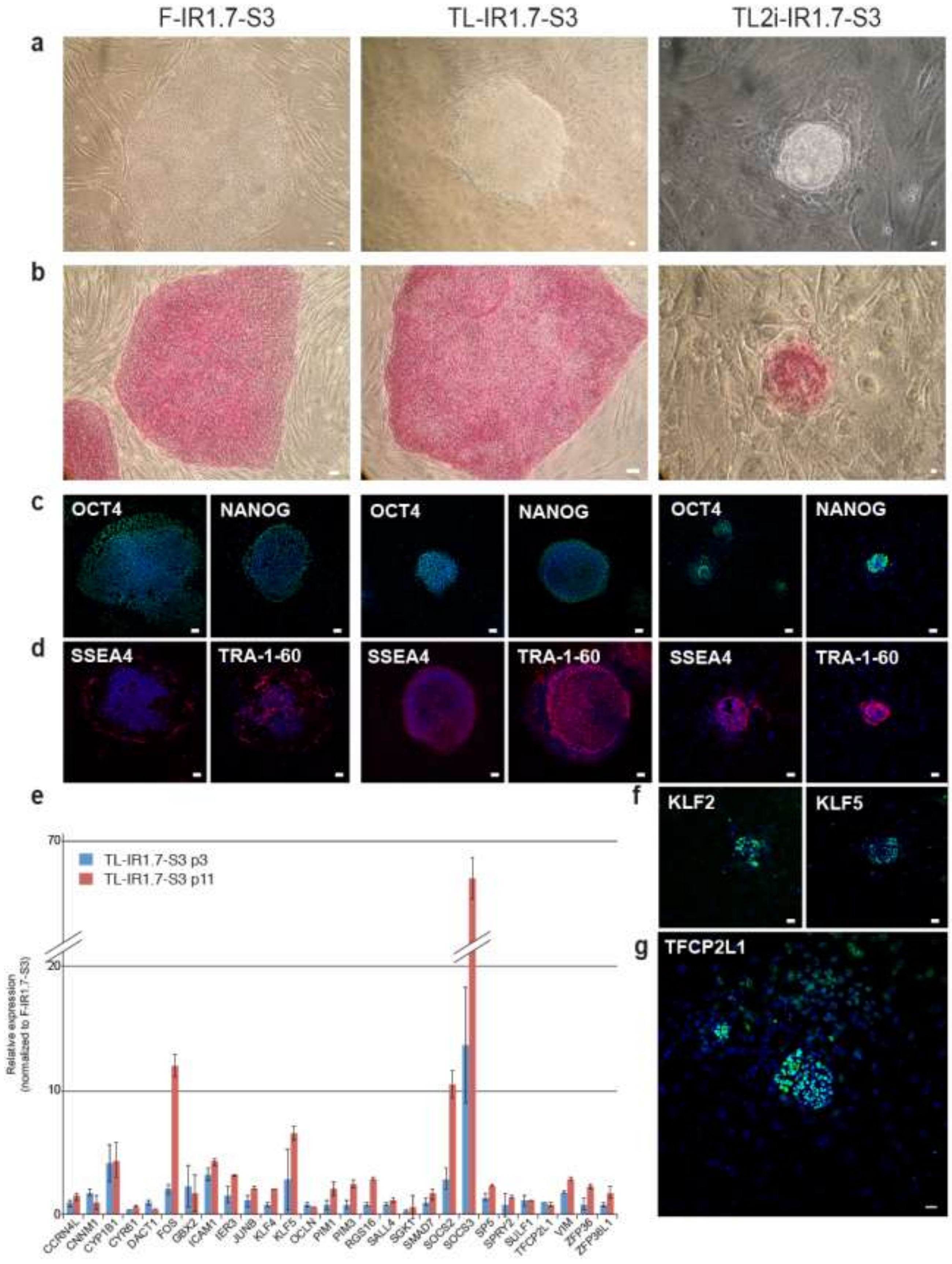


**Supplementary File 9: Reprogramming of human iPS cells to TL and TL2i states.** Human iPSCs (IR1.7 line, Ng et al., 2012) were infected with GAE-STAT3-ER$^{T2}$. F-IR1.7-S3 cells stably expressing STAT3-ER$^{T2}$ were isolated and subsequently reprogrammed to a TL state (TL-IR1.7-S3) by LIF and 4'OHT as evidenced by their morphology, AP staining, and immunolabeling for OCT4, NANOG, SSEA-4, and TRA-1-60 expression. In the next step, the TL-IR1.7-S3 cells were successfully reprogrammed to

TL2i-IR1.7-S3 cells after treatment with MEK and GSK3β inhibitors for 5 passages. The TL2i-IR1.7-S3 cells displayed the typical dome-shape morphology of TL2i cells and expressed OCT4, NANOG, SSEA4, TRA-1-60, TFCP2L1, KLF2, and KLF5 as previously described with TL2i-OS3 cells.

**(a)** Phase contrast. (**b**) Alkaline phosphatase activity. (**c**) Immunolabeling for OCT4 (left panel) and NANOG (right panel). (**d**) Immunolabeling for SSEA4 (left panel) and TRA-1-60 (right panel). (**e**) Histogram representation of the mRNA level (ΔCt) of STAT3 target genes in F-IR1.7-S3 and TL-IR1.7-S3 cells, at passage 3 and 10, after normalization to *β-actin* (ΔCt = 1; n = 3, mean ± s. d.) (**f**) Immunolabeling for KLF2 (left panel) and KLF5 (right panel). (**g**) Immunolabeling for TFCP2L1. Scale bars = 50 µm.

Ng, S.Y., Johnson, R., & Stanton, L.W. Human long non-coding RNAs promote pluripotency and neuronal differentiation by association with chromatin modifiers and transcription factors. *EMBO J* **31**, 522-533 (2012).

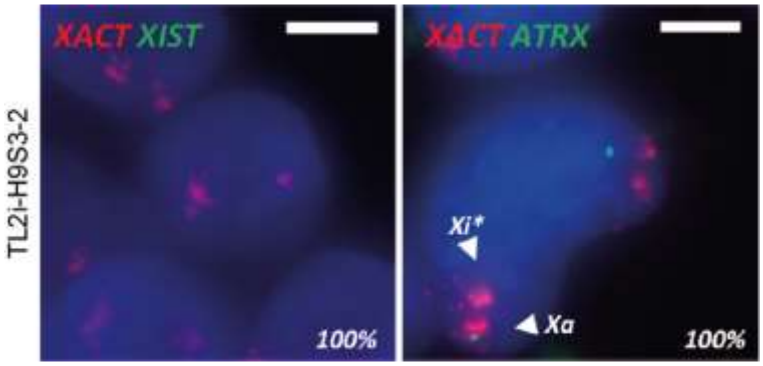


**Supplementary File 10: X-chromosome activity status in TL2i-H9 cells.** RNA-FISH analysis of *XACT* (in red), *XIST* and *ATRX* (in green) in TL2i-H9S3-2. Co-RNA FISH with *XACT* in *ATRX* probes (right panel) allows to distinguish between active X chromosome (Xa) and a partially reactivated X (Xi*). Scale bars correspond to 5 μm.

**a**

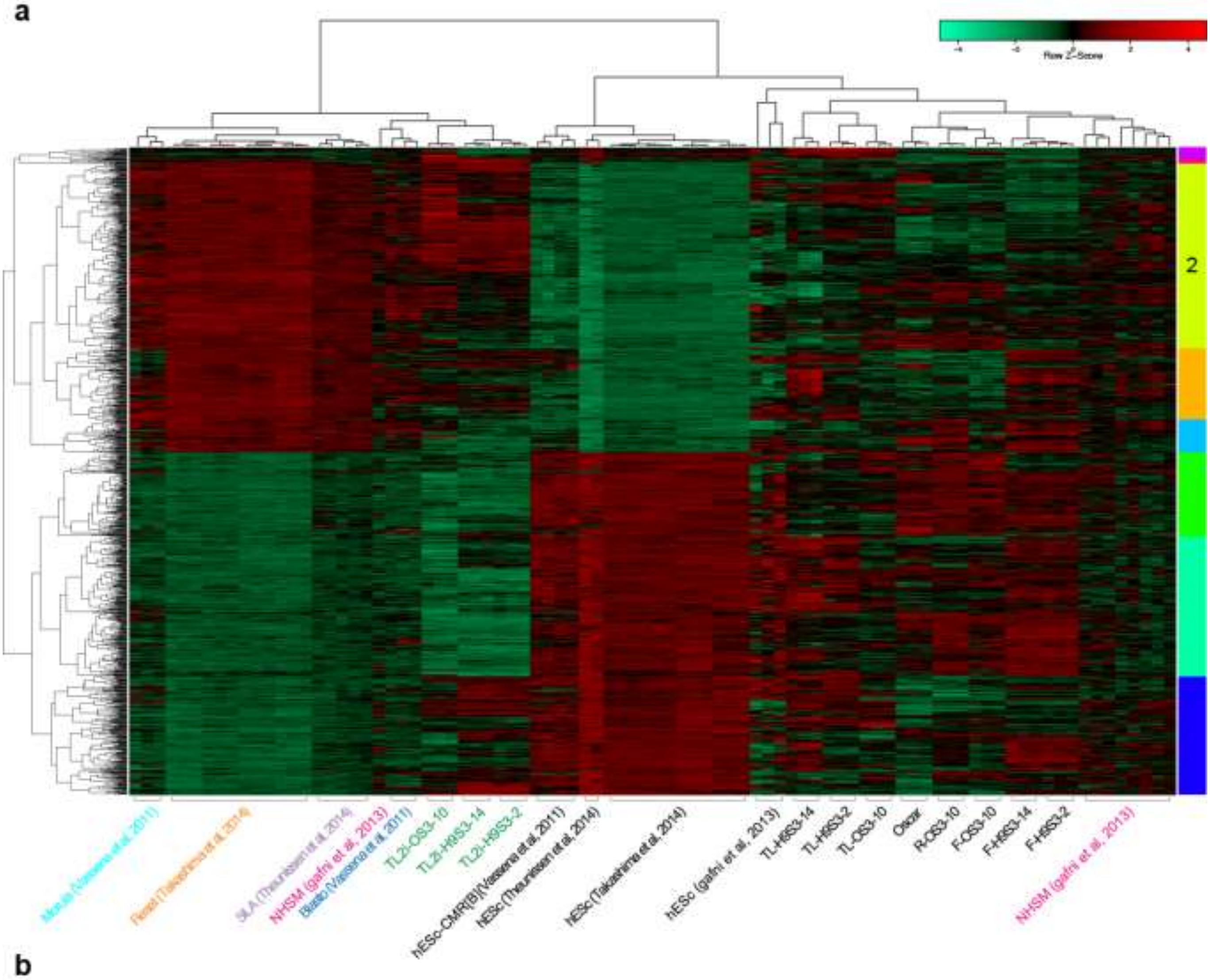


**b**

**Cluster 2**

| GO term | Description | P-value | FDR q-value | Enrichment (N, B, n, b) |
|---|---|---|---|---|
| GO:0006626 | protein targeting to mitochondrion | 4.36E-8 | 5.57E-4 | 12.02 (16480,43,287,9) |
| GO:0072655 | establishment of protein localization to mitochondrion | 1.45E-7 | 9.26E-4 | 10.55 (16480,49,287,9) |
| GO:0070585 | protein localization to mitochondrion | 2.08E-7 | 8.87E-4 | 10.13 (16480,51,287,9) |
| GO:0006839 | mitochondrial transport | 7.96E-6 | 2.54E-2 | 5.86 (16480,98,287,10) |
| GO:0050702 | interleukin-1 beta secretion | 2.06E-5 | 5.27E-2 | 43.07 (16480,4,287,3) |
| GO:0006457 | protein folding | 5.01E-5 | 1.07E-1 | 3.73 (16480,200,287,13) |
| GO:0050701 | interleukin-1 secretion | 1.01E-4 | 1.83E-1 | 28.71 (16480,6,287,3) |
| GO:0044238 | primary metabolic process | 1.93E-4 | 3.08E-1 | 1.22 (16480,7794,287,166) |
| GO:0008152 | metabolic process | 2.96E-4 | 4.2E-1 | 1.19 (16480,8840,287,183) |
| GO:0071409 | cellular response to cycloheximide | 3.02E-4 | 3.86E-1 | 57.42 (16480,2,287,2) |
| GO:0071704 | organic substance metabolic process | 3.26E-4 | 3.79E-1 | 1.21 (16480,8035,287,169) |
| GO:0044237 | cellular metabolic process | 3.53E-4 | 3.76E-1 | 1.22 (16480,7700,287,163) |
| GO:0006412 | translation | 5.13E-4 | 5.03E-1 | 3.12 (16480,221,287,12) |
| GO:0007126 | meiotic nuclear division | 5.5E-4 | 5.01E-1 | 5.84 (16480,59,287,6) |
| GO:0051085 | chaperone mediated protein folding requiring cofactor | 5.73E-4 | 4.88E-1 | 17.23 (16480,10,287,3) |
| GO:0071806 | protein transmembrane transport | 6.18E-4 | 4.93E-1 | 7.18 (16480,40,287,5) |
| GO:0071479 | cellular response to ionizing radiation | 6.94E-4 | 5.21E-1 | 7.00 (16480,41,287,5) |
| GO:0051084 | 'de novo' posttranslational protein folding | 9.64E-4 | 6.84E-1 | 6.53 (16480,44,287,5) |

**Supplementary File 11: Transcriptome meta-analysis of “naïve” human PSCs.**

(**a**) Hierarchical clustering of the transcriptome data from the compendium described in **Fig. 8a**, using Pearson correlation coefficient as a measure of distance between rows and Spearman correlation

coefficient as a measure of distance between columns. (**b**) Analysis of cluster 2 in the heatmap shown in **Sup. File 11a**, corresponding to probe sets upregulated in TL2i, Reset and 5iLA cells *vs* NHSM, F and TL cells. Gene ontology analysis of this cluster identified 18 cellular functions including (i) protein targeting to mitochondrion, (ii) establishment of protein localization to mitochondrion, (iii) protein localization to mitochondrion, and (iv) mitochondrial transport.

**Supplementary Table S4: List of primary antibodies.**

| Name | Supplier | Reference | Dilution |
|---|---|---|---|
| $\alpha$-ACTIN | Millipore | CBL171 | 1/200 |
| $\alpha$-ACTIN-HRP | Sigma Aldrich | A3854 | 1/10000 |
| DESMIN | DAKO | M0760 | 1/50 |
| FOXA2 | Millipore | 07-633 | 1/100 |
| GATA4 | Millipore | AB-4132 | 1/200 |
| KLF2 | Sigma | SAB1101046 | 1/100 |
| KLF4 | Stemgent | 09-0021 | 1/100 |
| KLF5 | Gifted Dr H. Ng | | 1/100 |
| KLF5 (WB) | AbCam | ab137676 | 1/1000 |
| NANOG | Peprotech | 500-p236 | 1/300 |
| NANOG | eBiosciences | 14-5768-82 | 1/100 |
| OCT4 | Santa Cruz | sc-5279 | 1/100 |
| OCT4 | Santa Cruz | sc-9081 | 1/200 |
| SSEA4 | Millipore | MAB4304 | 1/100 |
| STAT3 (WB) | BD | 610190 | 1/2000 |
| STAT3 | Cell Signaling | 07/2013 | 1/1300 |
| p-STAT3 (Ser 727) (WB) | BD | 612543 | 1/100 |
| p-STAT3 (Tyr 705) (WB) | Santa-Cruz | sc-8059 | 1/100 |
| p-STAT3 (Tyr 705) | Cell Signaling | 20/2013 | 1/100 |
| TFCP2L1 | Abcam | ab123354 | 1/200 |
| TFE3 | Thermo Sciences | PA5-21615 | 1/250 |
| TRA-1-81 | Millipore | MAB4381 | 1/100 |
| TRA-1-60 | Millipore | MAB4360 | 1/100 |
| TUJ1/B-III Tubulin | Millipore | MAB1637 | 1/200 |

## Supplementary Table S5: Primers for qRT-PCR and single cell qPCR

| Gene | Forward Primer (5'--3') | Reverse Primer (5'--3') |
|---|---|---|
| ACVR2A | GGCCAGCATCCATCTCTTGA | GCGTCGTGATCCCAACATTC |
| ACVR2B | ACCCGTGGATGAGTACATGC | CACTCCTCGATGGTCACACA |
| AIRE | CCCAGGCTCTCAACTGAAGG | GTCTGAATCCCGTTCCCGAG |
| BLIMP1 | GGCACCCTTGCCTACTGTAA | CTTCCTCCCTGGTTGTTTTG |
| BRA | CCTTGGAATGCCTGCCCATCCC | GCGCCGTTGCTCACAGACCAC |
| CCRN4L | ACCCACCCACCTATCAGGGTTATGC | GGCTGGTAGGCCAGGATTTCTTCC |
| CDH1 | ACGCATTGCCACATACACTC | TCGGGCTTGTTGTCATTCTG |
| CDH2 | TGTGACCGATAAGGATCAACCC | TAACCCGTCGTTGCTGTTTG |
| CER1 | CAGCCAGACTATAACCCACGAA | GCGGCTCCAGGAAAATGAAC |
| cFOS | AGCCAAATGCCGCAACCGGA | CGGTGAGCTGCCAGGATGAACT |
| cMYC | TTCGGGTAGTGGAAAACCAG | CAGCAGCTCGAATTTCTTCC |
| CNNM1 | GAAATTTCGGCACACCCTA | TGGCACTGGATGACTGTGAA |
| CYP1B1 | GACATCTTCGGCGCCAGCCAG | CAAGGCAGACGGTCCCTCCC |
| CYR61 | GTGAGGTGCGGCCTTGTGGAC | AGGAACCGCAGTACTTGGGCCG |
| DACT1 | CAGCCCCTTGGAGGCGACCTT | GTGGTGTGGAGAGGGAACGGC |
| DDX3 | ATGTGGCAGTGGAAAATGCG | AGGAATATAGCGCCCTTTGC |
| DNMT3A | CTTTAGCCGATTCGAGGGCT | TTTGCTCCAGGTGGGGTTTT |
| DNMT3B | GCTGTTTGTCTTGTGGCAGG | ACAGCTCAAGGAAGCGATCC |
| DNMT3L | TCTCGCTTCCTGGAGATGGA | CTGCTCCTTATGGCCGGAAT |
| DPPA3 | GGCGGAGTTCGTACGCATGAAAGA | GACACGCAGAAACTGCAGGGACA |
| DPPA5 | ACCTGAAAGATCCAGAGGTGT | CCGGCTTCATTGCATTGGCT |
| ESRRB | TGCCCTATGACGACAAGCTG | TGAGCGTCACAAACTCCTCC |
| FGF4 | CCAACAACTACAACGCCTACGA | CCCTTCTTGGTCTTCCCATTCT |
| FGF5 | CCATGCAAGTGCCAAGTTCACAGA | GGGGCTGCACCCTCGTTTGG |
| FOXD3 | ACAACCTCTCACTCAACGACTG | CTGCCGTTGTCGAACATGTC |
| GAPDH | GGCCCCCTCAAGGGCATCCT | GGGCCATGAGGTCCACCACC |
| GBX2 | CACCACGTCTACGGGCAAGAAC | AGCTGCTGATGCTGACTTCTGA |
| GDF3 | AGACTTATGCTACGTAAAGGAGCT | CTTTGATGGCAGACAGGTTAAAGTA |
| GP130 | ACACAGCAGTGGTATTGGGG | ACTGGACAGTGCTCGAAGTG |
| ICAM1 | CAGGGAATATGCCCAAGCTA | GAACCATGATTGCACCACTG |
| IER3 | TCTCGCGGGCACCGAAAGC | GCAGAAGACGATGGTGAGCAGC |
| IGF2R | TAATGTCTGCGGCACAATGC | AGTTTGGGTTTCTGCCTCAC |
| JAK1 | CCAGAACTGCCCAAGGACAT | CGCATCCTGGTGAGAAGGTT |
| JAK2 | CCACCCAACCATGTCTTCCA | CCATGCCGATAGGCTCTGTT |
| JAK3 | TTCACAAGATCCCCGCTGAC | AGCCCCGGTAAATCTTGGTG |
| JUNB | GAGAACGCGGGGCTGTCGAG | AGGCGTGTCCCTTGACCCCA |
| KLF2 | AGAGGGTCTCCCTCGATGAC | CTCGTCAAGGAGGATCGTGG |
| KLF4 | CCCTACCTCGGAGAGAGACC | GGATGGGTCAGCGAATTGGA |
| KLF5 | AGATGTTCGCTCGTGCAGTA | TCTGCCCTTTGGTTAACAGC |
| LEFTY1 | ACACCCTGGACCTTGGGG | CCAGTTCTCGGCCCACTT |
| LEFTY2 | CCCTGGACCTCAGGGACTAT | ACACTCGTAAGCCAGGAAGC |
| LHX2 | GCGATGCTGTTCCACAGTCT | GCATGGTCGTCTCGGTGTC |
| LIFR | ATATGCCCTTGGAGTGTGCC | CTCCACTCTTTGCGACCAGA |
| LIN28 | GAAGGGTTCCGGAGCTTGAA | ACAGTTGTAGCACCTGTCTCC |
| NANOG | CGCCCTGCCTAGAAAAGACA | GCCAGAGACGGCTTCTATCAA |
| NR0B1 | CCAAGCCATCAAGTGCTTTC | ATTTGCTGAGTTCCCCACTG |
| OCLN | TGAAGAGTACATGGCTGCTGCTGA | GCTTCCGGGGTTATGGTCCAAAGT |

| | | |
|---|---|---|
| OCT4 | ACATCAAAGCTCTGCAGAAAGAACT | CTGAATACCTTCCCAAATAGAACCC |
| OTX2 | GACCACTTCGGGTATGGACT | TGGACAAGGGATCTGACAGT |
| PECAM1 | CCAGTGTCCCCAGAAGCAAA | TCCGATGACAACCACTGCAA |
| PIM1 | GCCATCAAACACGTGGAGAA | GCCTAATGACGCCGGAGAAA |
| PIM3 | TGGTCGCTGGGCGTGCTTCT | TCCAGGGATGGGCCGCAATCT |
| PRDM14 | AGACGGTGTTTGGTGAAGTC | ACTTCACTGGCATTGACCAC |
| REX1 | GGAATGTGGGAAAGCGTTCGT | CCGTGTGGATGCGCACGT |
| RGS16 | AAGCTGGCCTCCAGGGCACAC | TCCTCGTCAGCTCGTGGGTCTC |
| SALL4 | ATCCACGAGCGGACTCACACTGG | GGCCAACTTCCTTCCACGGCG |
| SGK1 | CGGAATGTTCTGTTGAAGAATGTG | TGTCAGCAGTCTGGAAAGAGAAGT |
| SMAD7 | AGCAGCCACACTTCAAACT | GGTGCTTGGATTTCTGCTTC |
| SOCS2 | TGAGTGATGCTTCCCTTCCT | AGCTTGGTTCCTTCCCACTT |
| SOCS3 | TCCCCCCAGAAGAGCCTATTAC | TCCGACAGAGATGCTGAAGAGTG |
| SOX17 | TTGGAAGGCGTTGACCTTGGCAG | TTCATGCGCTTCACCTGCTTGC |
| SOX2 | GGGAAATGGGAGGGGTGCAAAAGAGG | TTGCGTGAGTGTGGATGGGATTGGTG |
| SP5 | GGGCCTGCAAGCCGTAGCCAT | AATGCCAGGGGCGAGTGCTT |
| SPRY2 | TGCCATACTACATGGCACAGA | TCTGTAACCCCTCATTTGCAG |
| STAT3 | TGATGCAGTTTGGAAATAATG | GCGCCTCAGTCGTATCTTTC |
| STELLA | GGCGGAGTTCGTACGCATGAAAGA | GACACGCAGAAACTGCAGGGACA |
| SULF1 | AGCAGTGCAACCCAAGACCTAAGA | TGTCTGCAGTGAGACGGGGCT |
| TBX3 | GGATGTCCAAAGTCGTCA | GCTGGTATTTGTGCATGGAGTTCA |
| TCF3 | ACAGACAAGGAGCTCAGTGAC | TCAAGACCTGAACCTCCGAAC |
| TDGF1 | GTGTAAATGCTGGCACGGTC | GGCAGATGCCAACTAGCATAA |
| TFCP2L1 | AGCAACCTGTCTGTGTACCAC | TGGCGATCTTCTCAATCAGC |
| VIM | CACTCCCTCTGGTTGATACCCACTC | TGCTGCACTGAGTGTGTGCAATTTT |
| ZFP36 | CTTCAGCGCTCCCACTCTCGG | CGTCAGGGCTCAGCGACAGGA |
| ZFP36L1 | ATTACCTCTTCAGCGCCAGA | AGACAGGTTTCCCCCAAAAC |
| ZIC1 | AAACTGGTTAACCACATCCGC | CTCAAACTCGCACTTGAAGG |
| ZIC4 | CCCAGCTCTGGCTACGATTC | GATGTAGCAGGCGCGAGAT |
